# Associations of Different Definitions of Prediabetes and Diabetes with All-Cause and Cause-Specific Mortality: A Nationally Representative Cohort Study

**DOI:** 10.1016/j.mmr.2026.100028

**Published:** 2026-04-22

**Authors:** Mei Zhang, Zixin Qiu, Yue Wang, Xiao Zhang, Shiyu Zhao, Chun Li, Zhenping Zhao, Shenhan Xiang, An Pan, Limin Wang, Maigeng Zhou, Kai Huang, Gang Liu

**Affiliations:** a National Center for Chronic and Noncommunicable Disease Control and Prevention, Chinese Center for Disease Control and Prevention, Beijing 102206, China; b Department of Nutrition and Food Hygiene, Hubei Key Laboratory of Food Nutrition and Safety, Ministry of Education Key Laboratory of Environment and Health, and State Key Laboratory of Environment Health (Incubating), School of Public Health, Tongji Medical College, Huazhong University of Science and Technology, Wuhan 430030, China; c Clinical Center for Human Genomic Research, Union Hospital, Huazhong University of Science and Technology, Wuhan, China; d Hubei Clinical Research Center of Metabolic and Cardiovascular Disease, Huazhong University of Science and Technology, Wuhan, 430022, China; e Hubei Key Laboratory of Metabolic Abnormalities and Vascular Aging, Huazhong University of Science and Technology, Wuhan, 430022, China; f Department of Epidemiology and Biostatistics, School of Public Health, Tongji Medical College, Huazhong University of Science and Technology, Wuhan 430030, China; g Center for Obesity and Diabetes Research, School of Public Health, Tongji Medical College, Huazhong University of Science and Technology, Wuhan 430030, China; h Renmin Hospital, Wuhan University, Wuhan, 430000, China; i State Key Laboratory of Metabolism and Regulation in Complex Organisms, Wuhan University, Wuhan, 430000, China

**Keywords:** diabetes, prediabetes, mortality, life expectancy, prospective study

## Abstract

**Background:**

The associations between different definitions of prediabetes and diabetes with mortality and life expectancy remain unclear. Clarifying these associations is essential for informing hyperglycemia management policies. We aimed to compare the prevalence of prediabetes and newly diagnosed diabetes across different glycemic indicators and diagnostic criteria, and to examine their associations with mortality and life expectancy.

**Methods:**

We analyzed data from 141,945 adults from a nationally representative cohort study in China. Cox proportional hazards regression models were used to estimate hazard ratios (HRs). Using fasting plasma glucose (FPG), 2-hour postload glucose (2hPG), and hemoglobin A1c (HbA1c) levels, prediabetes was defined according to the American Diabetes Association (ADA), World Health Organization (WHO), or International Expert Committee (IEC) criteria, and newly diagnosed diabetes according to the ADA criteria.

**Results:**

Prediabetes prevalence varied widely across glycemic indicators, ranging from 3.0% to 26.1%, while newly diagnosed diabetes prevalence ranged from 2.6% to 4.4%. Over a median follow-up of 9.0 years, a total of 6,924 deaths were documented. Compared with people with normoglycemia, prediabetes defined by FPG (either ADA or WHO criteria) was not significantly associated with increased risks of all-cause or cardiovascular disease (CVD) mortality (all *P*>0.05). In contrast, prediabetes defined by 2hPG or HbA1c (either ADA or IEC criteria) was each associated with higher risks of all-cause mortality (HRs: 1.13–1.23; all *P*<0.001) and CVD mortality (HRs: 1.12–1.25; all *P*<0.001). Prediabetes defined by 2hPG or HbA1c, but not FPG, was associated with 1.1–2.3 years reduction in life expectancy, with the largest loss observed for IEC HbA1c. In addition, diabetes defined by FPG, 2hPG or HbA1c was each significantly associated with higher risk of all-cause and CVD mortality (HRs: 1.25–1.51), and reduction in life expectancy (2.0–3.7 years). Furthermore, the 2hPG-based definition of prediabetes and diabetes was associated with mortality risk, independent of FPG and HbA1c levels.

**Conclusions:**

These findings suggest that reliance on FPG alone may fail to identify certain individuals at elevated mortality risk. In contrast, 2hPG and HbA1c provide additional prognostic information beyond FPG.

## 1 Introduction

Type 2 diabetes (T2D) represents a persistent global health challenge, with China experiencing the largest burden of prediabetes and diabetes worldwide [1]. In 2024, 148.0 million adults in China and were living with diabetes, constituting approximately 25% of the global diabetes burden [2]. Concurrently, prediabetes remains highly prevalent among Chinese adult population, defining a substantial large high-risk cohort for future progression to diabetes [3]. Both conditions, as disorders of glucose metabolism, are associated with a significant reduction in life expectancy and a increased risks of cardiovascular and cancer mortality [4], [5], [6]. Specifically, individuals with prediabetes exhibit a 13–30% elevated risk of cardiovascular events compared to normoglycemic individuals [7], while those with T2D face a substantially greater cardiovascular burden, with relative risks typically increased two- to fourfold [8]. Given the considerable and growing burden of dysglycemia in China [3], proactive screening and early intervention in this population are crucial to mitigate future disease complications and reduce the associated public health impact.

In contemporary guidelines, prediabetes and diabetes can be identified using any fasting plasma glucose (FPG), 2-hour postload glucose (2hPG) and hemoglobin A1c (HbA1c), or a combination of these measures [5], [6], [9]. FPG has emerged as the most widely adopted test in clinical setting, largely due to its practicality and lower cost [10], [11]. Although all three glycemic indicators offer clinic utility, the estimated prevalence of prediabetes and diabetes varies substantially depending on which criterion is applied. Furthermore, their associations with long-term mortality differ across indicators [12], [13], [14], [15], [16], [17]. Previous studies investigating these associations have reported inconsistent results, often limited by the assessment of only one or two glycemic measures, modest sample sizes, and restricted generalizability [12], [13], [14], [15], [16], [17]. Comprehensive evidence, particularly in East Asia populations, remains scarce [9], [18]. Additionally, while diabetes is uniformly defined according to established American Diabetes Association (ADA) thresholds, there is no international consensus on the optimal definition of prediabetes; five distinct sets of diagnostic cutoffs are currently recommended by major guideline bodies [5], [6], [9]. A clearer delineation of how these diagnostic criteria and corresponding biomarkers relate to mortality and life expectancy is essential to refine screening approaches, inform early intervention, and ultimately mitigate the disease burden attributable to dysglycemia.

To address these evidence gaps, the present study utilizes data from a nationally representative cohort in mainland China to systematically compare the prevalence of prediabetes and newly diagnosed diabetes as defined by various diagnostic criteria and glycemic indicators. Furthermore, it evaluates the associations of these differently defined glycemia states with all-cause and cause-specific mortality, and estimates their corresponding impacts on life expectancy.

## 2 Methods

### 2.1 Study Design and Participants

The China Chronic Disease and Risk Factors Surveillance (CCDRFS), established by the National Center for Chronic and Noncommunicable Disease Control and Prevention (NCNCD) of the Chinese Center for Disease Control and Prevention (China CDC), is a series of nationally representative cross-sectional surveys designed to assess the trends in the prevalence of noncommunicable diseases and related risk factors across mainland China[1], [3], [19]. The CCDRFS uses a multistage stratified random sampling strategy to ensure national representativeness, covering all 31 provinces, autonomous regions, and municipalities. Noninstitutionalized participants aged ≥ 18 years who had resided at their current residence for at least 6 months during the year preceding the survey were eligible for enrollment. This study utilizes data from the 2013 CCDRFS. Detailed information on data collection and covariates are summarized in Supplementary Methods. The 2013 CCDRFS and this study protocol were approved by the ethical review committee of the NCNCD. All participants signed the written informed consent. The 2013 CCDRFS achieved a response rate of 93.4%. From an initial pool of 156,063 participants with valid identification numbers. We excluded 14,118 individuals due to missing data on blood glycemic indicators or self-reported diabetes status, resulting in 141,945 participants were included in the association analyses. For the prevalence analysis, individuals with missing glycemic data were excluded, yielding a final analytical sample of 143,094 participants.

### 2.2 Assessment of Prediabetes and Newly Diagnosed Diabetes

Fasting blood samples were obtained after at least 10 hours of overnight fasting. FPG and 2hPG following a standardized 75-g oral glucose tolerance test were measured in accordance with a national protocol [1], [3]. Plasma glucose concentrations were determined using either the glucose oxidase or the hexokinase method, with all participating laboratories followed a centralized standardization and quality-control program to ensure inter-laboratory comparability [20]. HbA1c was measured from venous blood stored at –80°C and analyzed within 1 month at a central certified laboratory. Detailed information on the assay method and quality control are provided in the Supplementary Methods.

Diagnostic criteria for prediabetes differ slightly among major organizations, including the ADA, the World Health Organization (WHO), and the International Expert Committee (IEC) [5], [6], [9]. The ADA defines prediabetes as an FPG level of 5.6–6.9 mmol/L, a 2hPG level of 7.8–11.0 mmol/L, or a HbA1c level of 5.7%-6.4%. The WHO used a slightly higher FPG cutoff (6.1–6.9 mmol/L) with the same 2hPG range as the ADA’s, whereas the IEC recommends an HbA1c range of 6.0%–6.4%. For the purposes of this epidemiological analysis, newly diagnosed diabetes was defined as having no prior diagnosis of diabetes and meeting at least one of the following glycemic criteria at baseline: an FPG level ≥7.0 mmol/L, a 2hPG level ≥11.1 mmol/L after a 75-g oral glucose tolerance test, or an HbA1c level ≥6.5% [21]. Although this classification aligns broadly with clinical diagnostic standards, it differs from routine clinical practice, which typically involves confirmatory testing and stepwise diagnostic algorithms.

### 2.3 Ascertainment of Death

Mortality outcomes for all cohort participants were ascertained through automated linkage with the National Mortality Surveillance system, with follow-up extending through 31 December 2021. Death records were annually verified and coded in accordance with standardized quality control procedures[22]. The underlying causes of death were classified using the 10th revision of the International Statistical Classification of Diseases and Related Health Problems (ICD-10). CVD mortality included codes I00–I99, and cancer mortality included codes C00–C97.

### 2.4 Statistical Analyses

Weighted estimates of the prevalence of prediabetes and newly diagnosed diabetes by different definitions were calculated. The statistical weights consisted of two components: sampling weights, derived from the inverse of each participant’s selection probability to account for the complex survey design, and post-stratification weights, calibrated to align the sample demographics with the age- and sex-specific structure of the 2010 Chinese national census population. Sample characteristics were reported as mean ± standard deviations (SD) for continuous variables, and percentages for categorical variables. Cox proportional hazard models were used to estimate adjusted hazard ratios (HRs) of all-cause and cause-specific mortality associated with the different definitions, with normoglycemia as the reference group. Person-years were calculated from baseline to the date of death or censoring date. Schoenfeld residuals were used to test the proportional hazards assumption, and no violation was observed. HRs and 95% confidence intervals (CIs) were adjusted for residence area, age, sex, education level, household income, smoking, alcohol consumption, physical activity, BMI, red meat intakes, vegetable and fruit intakes, hypertension, dyslipidemia, and self-reported CVD and cancer. In addition to the covariables mentioned above, we included baseline glycemic indicators (continuous variable) in the mutually adjusted model to compare the strength of associations with mortality outcomes. Prior to mutual adjustment, collinearity diagnostics were assessed. Variance inflation factors (VIFs) for FPG, HbA1c, and 2hPG were 1.89, 1.69, and 1.81, respectively, and below commonly used thresholds for problematic multicollinearity (i.e., VIF >5). A series of sensitivity analyses were performed, including Fine–Gray competing-risks models for CVD and cancer mortality, and survey-weighted Cox models accounting for the complex survey design. To account for potential regression dilution bias arising from single baseline glycemic measurements, we conducted sensitivity analyses applying regression dilution correction using previously published reliability estimates (intraclass correlation coefficients or repeated-measure correlations) for each glycemic indicator [23], [24], [25]. Specifically, log-transformed HRs were divided by the corresponding reliability estimates to obtain corrected estimates. Further details are provided in the Supplementary Methods. We explored potential nonlinear relationships between glycemic levels and mortality risk using restricted cubic spline models. Missing data (all missing values<5%) were coded as a missing indicator category for categorical variables and with median values for continuous variables.

Stratified analyses were conducted by age (<65, ≥65 years), sex (males, females), residence area (urban, rural), and hypertension (yes, no). Potential effect modifications were examined by testing the corresponding multiplicative interaction terms. To quantify the reduction in life expectancy associated with different definitions of prediabetes and newly diagnosed diabetes, we used life tables. We built the life table starting at age 40 years and ending at age 100 years with the following 3 estimates to calculate the cumulative survival from 40 years onward: [1] sex- and age-specific all-cause mortality rates from the Global Burden of Disease 2019; [2] constant HRs for mortality associated with each glycemic exposure group estimated from the CCDRFS; and [3] sex- and age-specific population prevalences of the exposure groups. These life expectancy estimates reflect population-level, model-based summaries rather than individual-level predictions. Details of the methods used to estimate life expectancy are provided in the Supplementary Methods. By applying Arriaga’s decomposition method, we estimated the cause-specific contributions to the life expectancy difference between people with (pre)diabetes and people with normoglycemia to identify the key contributors to the overall reduction in life expectancy due to cause-specific mortality differences[26]. To address potential extrapolation beyond the 90-year upper age limit, we conducted sensitivity analyses by constructing the life table from age 40 to 90 years and applying competing-risk models for cause-specific mortality.

## 3 Results

**Fig. 1:**
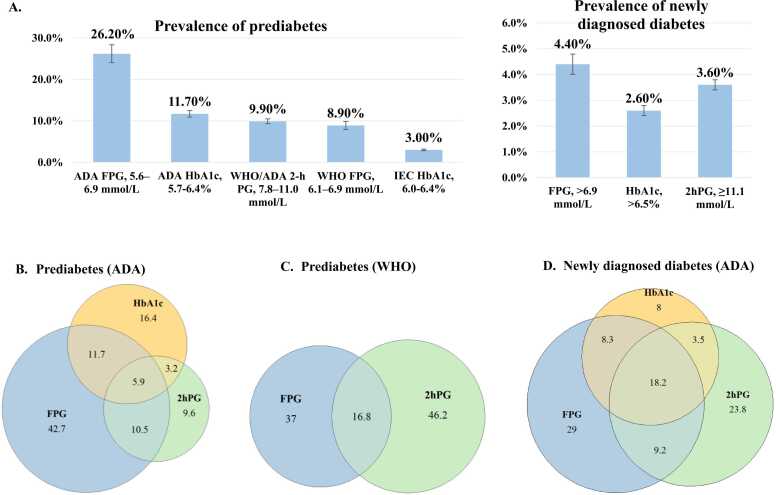
Prevalence of prediabetes and newly diagnosed diabetes in the Chinese population Aged ≥18 years by different definitions in 2013. A) Prevalence of prediabetes and diabetes by different definitions. B) The percentage of prediabetes by different glycemic indicators (ADA criteria). C) The percentage of prediabetes by different glycemic indicators (WHO criteria). D) The percentage of newly diagnosed diabetes by different glycemic indicators (ADA criteria). FPG, fasting plasma glucose; 2hPG, 2-hour postload glucose.

The study enrolled a cohort of 141,945 participants aged 18 years or older, with 42.9% being male. The median age was 50.9 years (interquartile range: 41.8–61.1). Different definitions of prediabetes and diabetes yield varying prevalence estimates. The prevalence of prediabetes was 36.2% (95% CI: 34.3%, 38.0%) using ADA criteria, 16.4% (15.3%, 17.4%) using WHO criteria, and 3.0% (2.8%, 3.2%) using IEC criteria. Moreover, the prevalence varied by glycemic indicator, being highest with ADA FPG (26.2%) and lowest with IEC HbA1c criteria (3.0%) (Fig. 1**A**). For newly diagnosed diabetes, the total prevalence was 6.8% (6.4%, 7.2%), with 4.4% (4.1%, 4.8%) defined by FPG, 2.6% (2.4%, 2.8%) by HbA1c, and 3.6% (3.3%, 3.8%) by 2hPG. These diversities were similar between males and females (Figure S1). Across all diagnostic definitions, the prevalence of prediabetes increased progressively with age (Figure S1).

Different definitions identify distinct prediabetes populations, with limited overlap. Based on ADA definition, FPG defined the largest proportion of prediabetes (70.8%), and only 5.9% were identified by all three definitions (Fig. 1**B**). The overlap between WHO FPG-defined prediabetes and WHO 2hPG-defined prediabetes was 16.8% (Fig. 1**C**). Compared with prediabetes identified by FPG, 2hPG or HbA1c-defined prediabetes were more likely to be older and female, with lower education and household income (Table S1). IEC HbA1c-defined prediabetes had the highest prevalence of overweight/obesity, hypertension and hyperlipidemia, and the lowest excessive alcohol drinker (Table S1). Among newly diagnosed diabetes, the largest proportion was identified by FPG (64.7%), and only 18.2% met all three criteria (Fig. 1**D**). Diabetes identified by HbA1c were more likely to be female, with the highest education level and prevalence of overweight/obesity and hyperlipidemia (Table S2). 2hPG-defined diabetes was the oldest and had the highest prevalence of hypertension (Table S2).

**Fig. 2:**
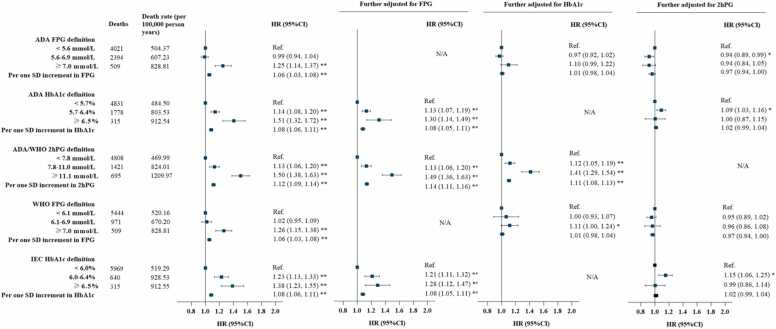
Number of deaths, all-cause mortality rates, and adjusted hazard ratios by different definitions of prediabetes and newly diagnosed diabetes**.** The HRs were adjusted for age (five-year group), sex (male, or female), residence (urban, or rural), education (junior high school and below, high school, college and above), household income (<¥20,000/year, ≥¥20,000/year, or not answer/don’t know), smoking (never smoked, past smoker, or active smoker), alcohol consumption (excessive, rare/nondrinker), physical activity (<150 min/week, or ≥150 min/week), red meat intakes (<100 g/day, or ≥100 g/day), vegetable and fruit intakes (<400 g/day, or ≥400 g/day), body mass index (<18.5, 18.5-23.9, 24-27.9, ≥28 kg/m^2^), hypertension (yes, or no), dyslipidemia (yes, or no) and self-reported CVD (yes, or no) and cancer (yes, or no). FPG, fasting plasma glucose; 2hPG, 2-hour postload glucose.

During 1,252,893 person-years of follow-up (median [interquartile range] follow-up: 9.0 [8.8, 9.3] years), there were 6924 deaths recorded, including 2327 CVD deaths and 1673 cancer deaths. In multivariable-adjusted Cox proportional hazard models, prediabetes defined by 2hPG or HbA1c was associated with higher risks of all-cause mortality (HRs ranged from 1.13 to 1.23; all *P* <0.001), with IEC HbA1c definition showing the largest HR, compared with normoglycemic individuals (Fig. 2). Notably, prediabetes diagnosed by FPG, of either ADA or WHO criteria, was not associated with the risk of all-cause mortality (HRs ranged from 0.99 to 1.02; all *P* >0.05) (Fig. 2). Similar patterns were observed for CVD mortality (Tables S3), while none of the prediabetes definitions were significantly associated with cancer mortality (Tables S4). Additionally, all diabetes definitions, including FPG, HbA1c or 2hPG, were associated with the increased risk of all-cause mortality (HRs ranged from 1.25 to 1.51; all *P* <0.001) and CVD mortality (HRs ranged from 1.25 to 1.44; all *P* <0.001) in individuals with newly diagnosed diabetes (Fig. 2 and Tables S3). Newly diagnosed diabetes defined by 2hPG, but not by FPG or HbA1c, was associated with cancer mortality (HR 1.49 [95% CI 1.26, 1.77]) (Tables S4). The results remained materially unchanged across all sensitivity analyses, including Fine–Gray competing-risks analyses for CVD and cancer mortality (Table S5), survey-weighted Cox models accounting for the complex survey design (Table S6), and sensitivity analyses addressing potential regression dilution (Table S7). To compare the strength of these associations with mortality risk, we included the baseline FPG, HbA1c and 2hPG levels in the model and performed mutual adjustments. Notably, only 2hPG-defined prediabetes remained significantly associated with an increased risk for all-cause and CVD mortality after adjustment for FPG or HbA1c levels (Fig. 2 **and** Tables S3**-4**). Similar independent associations were observed for 2hPG-defined newly diagnosed diabetes, which remained associated with all-cause, CVD, and cancer mortality after accounting for the other glycemic measures (Fig. 2 **and** Tables S3**-4**). Multivariable-adjusted restricted cubic spline analyses showed that only 2hPG was positively associated with all-cause, CVD and cancer mortality in a linear dose-response relationship (*P*_*nonlinear*_ >0.05), while both FPG and HbA1c displayed J-shaped associations with all-cause and CVD mortality respectively (*P*_*nonlinear*_ <0.05) (Figure S2). The J-shaped associations were largely preserved after excluding participants who died within the first three years of follow-up or who had a history of CVD or cancer at baseline (Figure S3).

**Fig. 3:**
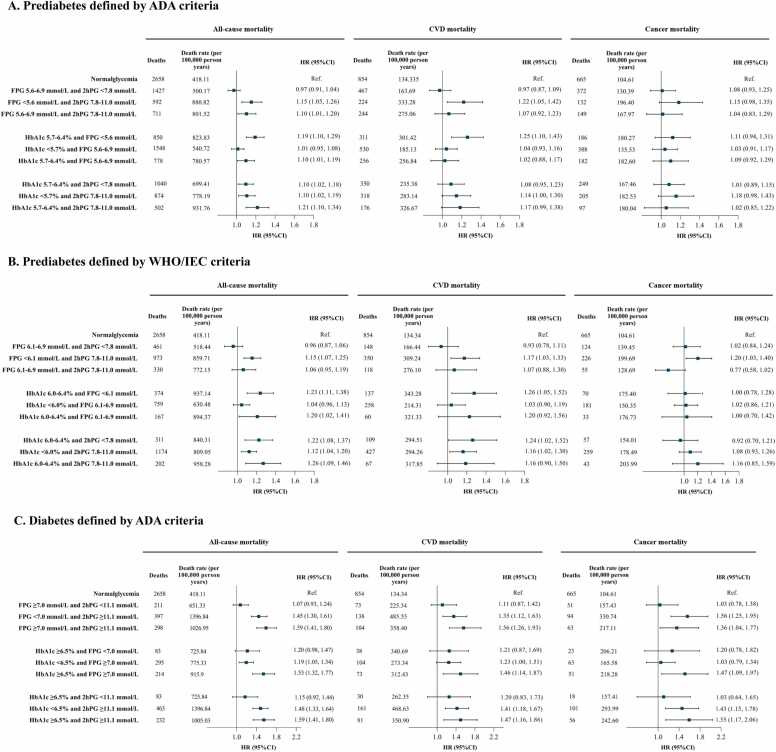
Number of deaths, all-cause and cause-specific mortality rates, and adjusted hazard ratios by different definitions of prediabetes and newly diagnosed diabetes. The HRs were adjusted for age (five-year group), sex (male, or female), residence (urban, or rural), education (junior high school and below, high school, college and above), household income (<20,000CNY/year, ≥20,000CNY/year, or not answer/don’t know), smoking (never smoked, past smoker, or active smoker), alcohol consumption (excessive, rare/nondrinker), physical activity (<150 min/week, or ≥150 min/week), red meat intakes (<100 g/day, or ≥100 g/day), vegetable and fruit intakes (<400 g/day, or ≥400 g/day), body mass index (<18.5, 18.5-23.9, 24-27.9, ≥28 kg/m^2^), hypertension (yes, or no), dyslipidemia (yes, or no) and self-reported CVD (yes, or no) and cancer (yes, or no). FPG, fasting plasma glucose; 2hPG, 2-hour postload glucose. Given the limited sample size, mutually exclusive phenotype analyses were conducted pairwise, focusing on two glycemic indicators at a time.

Fig. 3 presents the mortality risk associated with prediabetes and newly diagnosed diabetes based on combined criteria using FPG, HbA1c, and 2hPG, as defined by the ADA, WHO, and IEC. No significant increase in all-cause mortality risk was observed in individuals with prediabetes who met the FPG definition but did not meet the HbA1c (HRs ranged from 1.01 to 1.04; all *P* >0.05) or 2hPG definition (HRs ranged from 0.96 to 0.97; all *P* >0.05) (Fig. 3**A-B**). Additionally, individuals with prediabetes who met 2hPG definition had a significantly increased risk of all-cause mortality, regardless of whether they also met the HbA1c definition (HRs ranged from 1.10 to 1.26; all *P* <0.05) (Fig. 3**A-B**). The same results were observed among people with newly diagnosed diabetes defined by 2hPG (HRs ranged from 1.45 to 1.59; all *P* <0.05) (Fig. 3**C**). The results for CVD mortality were generally similar in direction. However, several subgroup estimates (e.g., the combined abnormal categories) did not reach statistical significance, probably due to the relatively small number of CVD deaths.

**Fig. 4:**
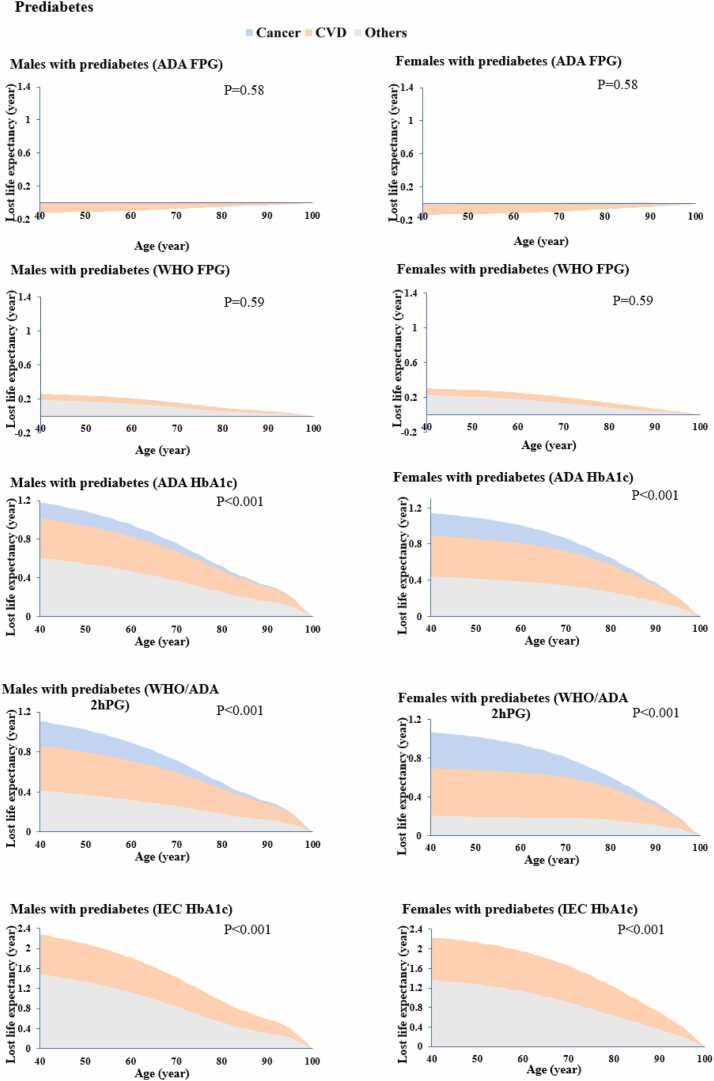
Estimated years of life lost attributable to increased deaths from cardiovascular disease, cancer, and other causes in people with prediabetes by different definitions. The HRs applied to estimate life expectancy were adjusted for age (five-year group), sex (male, or female), residence (urban, or rural), education (junior high school and below, high school, college and above), household income (<¥20,000/year, ≥¥20,000/year, or not answer/don’t know), smoking (never smoked, past smoker, or active smoker), alcohol consumption (excessive, rare/nondrinker), physical activity (<150 min/week, or ≥150 min/week), red meat intakes (<100 g/day, or ≥100 g/day), vegetable and fruit intakes (<400 g/day, or ≥400 g/day), body mass index (<18.5, 18.5-23.9, 24-27.9, ≥28 kg/m^2^), hypertension (yes, or no), dyslipidemia (yes, or no) and self-reported CVD (yes, or no) and cancer (yes, or no). FPG, fasting plasma glucose; 2hPG, 2-hour postload glucose.

Compared with normoglycemic individuals, 2 hPG and HbA1c-defined prediabetes were associated with a significant reduction in life expectancy (1.1-2.3 years). Among them, IEC HbA1c-defined prediabetes displayed the largest reduction (2.3 years for males; 2.2 years for females), followed by those defined by 2hPG (1.1 years for males and females) (Fig. 4**)**. In contrast, FPG-defined prediabetes was not associated with life expectancy loss (*P* >0.05). All definitions of newly diagnosed diabetes were associated with the reduction in life expectancy, with the most pronounced reduction observed under the 2hPG definition (up to 3.7 years) (Figure S4). Notably, CVD and other non-cancer deaths were the leading contributors to life-expectancy loss associated with prediabetes, with contributions varying by definition (35%–46% for CVD deaths and 19%–65% for other non-cancer causes). CVD deaths accounted for the largest proportion of life-expectancy loss associated with diabetes (35%–47%). The sensitivity analyses using a life table constructed from age 40 to 90 years and competing-risk models yielded results similar to the primary analyses (Figure S8).

In age-stratified analyses, 2hPG-defined prediabetes was associated with a higher relative risk of all-cause mortality among participants younger than 65 years, whereas the association was attenuated and not statistically significant among those aged 65 years or older (*P* for interaction = 0.03).Similar results were also observed in 2hPG-defined diabetes (HR 1.75 [95% CI: 1.53, 2.00] vs 1.37 [95% CI: 1.23, 1.52], *P* for interaction =0.02) (Table S5). No significant interaction was observed between different definitions and sex, residence area, or hypertension history (Tables S9**-11**).

## 4 Discussion

Based on a nationally representative cohort, we comprehensively compared definitions of prediabetes and diabetes across three glycemic indicators (FPG, 2hPG, and HbA1c) and among international organizations to provide an integrated evaluation of their impact on prevalence estimates and risk stratification. Our results revealed that different definitions for prediabetes and diabetes can yield varying estimates of its prevalence and identify distinct subsets of individuals at risk. Notably, 2hPG- or HbA1c-defined prediabetes was associated with higher all-cause and CVD mortality and shorter life expectancy, whereas FPG-defined prediabetes showed no significant association. These findings could inform more targeted approaches to defining prediabetes and diabetes in screening, potentially improving the prevention of hyperglycemia-related mortality.

In China, the prevalence of prediabetes varied substantially depending on the diagnostic threshold applied. Prediabetes defined by ADA FPG (26.2%) or HbA1c (11.7%) was almost two or three times higher than those defined by WHO FPG (8.9%) or IEC HbA1c (3.0%). Similar patterns were also observed in U.S. data, including both FPG (ADA 26.2% *vs* WHO 7.0%) and HbA1c (ADA 19.6% vs 6.2%) [27], [28]. Moreover, although ADA FPG definition identifies the majority of people with prediabetes, more than one third of HbA1c or 2hPG-defined prediabetes were classified as normoglycemia based on the FPG definition.

To date, the association between different glycemic indicators and mortality risk remains inconclusive. While several studies reported an increased risk of all-cause or CVD mortality among individuals with impaired fasting glucose (IFG), others did not find significant associations[7], [29], [30]. Some evidence suggested that 2hPG-defined (pre)diabetes was more strongly associated with mortality than FPG or HbA1c-defined (pre)diabetes[31], [32], [33]. In contrast, a UK study reported that 2hPG-defined diabetes only predicted cardiovascular risk when HbA1c was also elevated, raising questions about the added value of 2hPG in risk prediction[12]. More recently, a European study reported that sustained prediabetes—defined using repeated measurements, irrespective of the glycemic marker—was associated with increased cardiovascular and mortality risk even without progression to diabetes [34]. Differences across studies may reflect variations in population characteristics, follow-up duration, covariate adjustment, and whether prediabetes was defined longitudinally or at a single baseline assessment. Notably, most existing evidence was derived from Western populations and primarily focused on comparisons between only two glycemic indicators at a time. Given these limitations and the uncertain generalizability of prior findings to East Asian populations, our study provides important complementary evidence by examining all possible combinations of FPG, 2hPG, and HbA1c using data from a large, nationally representative Chinese cohort.

In the present study, we did not observe a significant association of FPG-defined prediabetes (of either ADA or WHO criteria) with increased risk of all-cause, CVD and cancer mortality in China. In contrast to FPG, 2hPG and HbA1c definition (of either ADA or WHO/IEC criteria) were associated with increased risk of all-cause and CVD mortality compared with normoglycemic individuals, respectively. These findings illustrated that relying solely on FPG definition can overlook the mortality risk of individuals with increased 2hPG or HbA1c but normal FPG levels. These implications are significant, especially in clinical settings where FPG is the primary method used for hyperglycemia screening[10].

European Diabetes Epidemiology Group recommends reassessing the current FPG cutoff for defining non-diabetic hyperglycemia, expressing concerns that a lower threshold could result in overdiagnosis and unnecessary treatment for individuals who may not be at significant risk[35]. Our results also support this recommendation. Furthermore, considering that the cutoff for defining prediabetes are mainly based on the studies conducted in Western countries, especially Europe and North America, while Asians typically have higher FPG levels than Europeans due to differences in genetic background, diet, and lifestyle [36], [37], the FPG cutoff for prediabetes in Asians need to be carefully explored in the future.

Our findings provide support for the potential added value of incorporating 2hPG and HbA1c measurements in risk stratification. Similarly, evidence from several large population-based studies have shown that dysglycemia defined by elevated 2hPG, but not FPG alone, is strongly associated with mortality risk [14], [17]. Other studies further demonstrated that 2hPG provides risk information independent of fasting glucose and HbA1c[38]. In parallel, HbA1c has been shown to identify a broader subgroup of individuals with elevated cardiometabolic risk and to be more strongly associated with CVD risk and mortality than FPG [39], [40].

As guidelines increasingly prioritize cardiometabolic screening, HbA1c is likely to become the primary test for diagnosing prediabetes, replacing OGTT [21]. This alteration raises the concern of whether this shift in diagnostic criteria could lead to missing individuals at high risk for mortality. Consistent with this, our data illustrate that the excess mortality risk of HbA1c-defined prediabetes can be primarily attributed to the postprandial hyperglycemia. This issue may be particularly relevant in Asian populations, where isolated impaired glucose tolerance is common [41], [42], [43], reflecting a pathophysiological profile characterized by relatively preserved hepatic insulin sensitivity, moderate to severe insulin resistance in skeletal muscle, and a pronounced defect in late-phase insulin secretion [44]. Additionally, recent evidence indicates that genetically determined hemoglobin variants prevalent among individuals of Asian ancestry can systematically lower HbA1c, potentially delaying the identification of dysglycemia [45]. Together, these findings emphasize the importance of the 2h OGTT in identifying high-risk individuals who may be missed by HbA1c-based screening strategies, particularly in Asian populations.

Although our findings indicate that 2hPG provide prognostic information beyond FPG and HbA1c, the feasibility of widespread OGTT remains limited in population-based and routine clinical settings because of time, cost, and participant burden. Universal OGTT screening is therefore unlikely to be practical. Rather than replacing FPG–based strategies, selective use of 2hPG or HbA1c among individuals with normal fasting glucose but elevated cardiometabolic risk may represent a pragmatic compromise.

In this study, prediabetes was not significantly associated with cancer mortality, a finding consistent with several prior cohort studies reporting weak or null associations for prediabetes [46], [47], [48], in contrast to the more consistently observed associations for overt diabetes [49], [50]. The absence of a clear association may reflect the relatively mild and heterogeneous nature of dysglycemia at the prediabetes stage, as well as potential exposure misclassification due to a single baseline measurement, limited numbers of cancer deaths, and heterogeneity across cancer types. Further studies with repeated glycemic measurements, longer follow-up, and cancer subtype–specific analyses are needed to clarify the relationship between prediabetes and cancer mortality.

The strength of this study includes the use of a large, nationally representative sample of the general population in China. The study adheres to rigorous protocols for assessing glycemia and related risk factors, employing standardized procedures and trained personnel. Additionally, the inclusion of all three key glycemic indicators is a significant advantage, as few studies have data on all three markers simultaneously. Several limitations of this study should be acknowledged. First, glycemic status was assessed only at baseline, and changes during follow-up were not captured, which may have introduced non-differential misclassification and attenuated associations toward the null. Future studies incorporating repeated glycemic measurements are warranted to evaluate mortality risks associated with persistent prediabetes and distinct glycemic trajectories, such as progression to diabetes or reversion to normoglycemia. Second, the lack of fasting insulin measurements limited our ability to directly assess insulin resistance, which may underline differences across glycemic markers. Future studies with insulin-related biomarkers are needed. Third, diabetes type could not be distinguished; however, given that incident cases occurred in adults from a population-based cohort, most cases are likely to be type 2 diabetes, and any potential misclassification would not materially affect the main findings. Fourth, although our results, consistent with previous studies [51], [52], suggested that prediabetes and diabetes were associated with higher mortality among younger participants than older participants, the age-stratified interaction analyses were exploratory and involved multiple subgroup comparisons, and further studies are needed for confirmation. Fifth, despite extensive adjustment for a number of demographic and clinical factors, we could not fully rule out the role of residual and unmeasured confounding by factors, such as medication use during follow-up, healthcare utilization patterns, and conditions affecting HbA1c validity (e.g., chronic kidney disease or anemia), etc, in our findings. These factors are more common among individuals with higher baseline health risk and greater clinical contact, and may be associated with either improved or worsened prognosis depending on the specific context. As a result, residual confounding related to these factors could bias effect estimates in either direction.

## 5 Conclusions

In a nationally representative cohort study, we found that prediabetes defined by 2hPG and HbA1c, but not FPG, were associated with increased risks of all-cause and CVD mortality in Chinese individuals. These findings suggest that reliance on FPG alone may fail to identify certain individuals at elevated mortality risk. In contrast, 2hPG and HbA1c provide additional prognostic information beyond FPG. From a clinical and public health perspective, targeted use of OGTT and HbA1c, particularly among high-risk individuals, may improve risk stratification and support earlier, more effective preventive interventions.

## Role of the Funding Source

The study was supported by the National Natural Science Foundation of China (research grant 82325043), Major Program of the National Natural Science Foundation of China (research grant 82495174) and National Key R&D Program of China (research grant 2023YFC2506504). The funder of the study had no role in study design, data collection, data analysis, data interpretation, or writing of the report.

## Author Contributions

MZ, ZQ, YW, XZ and SZ contributed equally to this study. GL, KH, LW, and MZ conceived and designed the study and took responsibility for the integrity of the data and the accuracy of the data analysis. ZQ, YW, KH, GL, and MZ drafted the manuscript. ZQ, YW, SZ and MZ did the analysis, and all authors critically revised the manuscript for important intellectual content and gave final approval for the version to be published. MZ, XZ, and SZ completed the follow-up work. XZ, HX and SZ accessed and verified the underlying data. LM, CL and ZZ give administrative, technical, or material support. All authors agree to be accountable for all aspects of the work in ensuring that questions related to the accuracy or integrity of any part of the work are appropriately investigated and resolved. All authors had full access to all the data in the study and had final responsibility for the decision to submit for publication.

## Competing interests

No potential conflicts of interest relevant to this article were reported.

## Data Availability

As subsequent follow-up investigations are still in progress, data collected for the study, including individual participant data, will not be made available to others. When all follow-up investigations are finished, data might be made available on request via email from the corresponding authors.

## Footnotes

Appendix A Supplementary data associated with this article can be found in the online version at doi:10.1016/j.mmr.2026.100028.

## References

[1] Wang L., Gao P., Zhang M., Huang Z., Zhang D., Deng Q. (2017). Prevalence and Ethnic Pattern of Diabetes and Prediabetes in China in 2013. Jama.

[2] Genitsaridi I., Salpea P., Salim A., Sajjadi S.F., Tomic D., James S., et al. Idf Diabetes Atlas: Global, Regional and National Diabetes Prevalence Estimates for 2024 and Projections for 2050.

[3] Wang L., Peng W., Zhao Z., Zhang M., Shi Z., Song Z. (2021). Prevalence and Treatment of Diabetes in China, 2013-2018. JAMA.

[4] Ahmad E., Lim S., Lamptey R., Webb D.R., Davies M.J. (2022). Type 2 diabetes. Lancet (London, England).

[5] Echouffo-Tcheugui J.B., Perreault L., Ji L., Dagogo-Jack S. (2023). Diagnosis and Management of Prediabetes: A Review. Jama.

[6] Echouffo-Tcheugui J.B., Selvin E. (2021). Prediabetes and What It Means: The Epidemiological Evidence. Annual review of public health.

[7] Cai X., Zhang Y., Li M., Wu J.H., Mai L., Li J. (2020). Association between prediabetes and risk of all cause mortality and cardiovascular disease: updated meta-analysis. BMJ.

[8] Harding J.L., Pavkov M.E., Magliano D.J., Shaw J.E., Gregg E.W. (2019). Global trends in diabetes complications: a review of current evidence. Diabetologia.

[9] Zimmet P., Alberti K.G., Magliano D.J., Bennett P.H. (2016). Diabetes mellitus statistics on prevalence and mortality: facts and fallacies. Nature reviews Endocrinology.

[10] (2023). Global variation in diabetes diagnosis and prevalence based on fasting glucose and hemoglobin A1c. Nature medicine.

[11] (1997). Report of the Expert Committee on the Diagnosis and Classification of Diabetes Mellitus. Diabetes care.

[12] Tabak A.G., Brunner E.J., Lindbohm J.V., Singh-Manoux A., Shipley M.J., Sattar N. (2022). Risk of Macrovascular and Microvascular Disease in Diabetes Diagnosed Using Oral Glucose Tolerance Test With and Without Confirmation by Hemoglobin A1c: The Whitehall II Cohort Study. Circulation.

[13] (2015). Effects of diabetes definition on global surveillance of diabetes prevalence and diagnosis: a pooled analysis of 96 population-based studies with 331,288 participants. The lancet Diabetes & endocrinology.

[14] Meigs J.B., Nathan D.M., D'Agostino R.B., Wilson P.W. (2002). Fasting and postchallenge glycemia and cardiovascular disease risk: the Framingham Offspring Study. Diabetes care.

[15] Barry E., Roberts S., Oke J., Vijayaraghavan S., Normansell R., Greenhalgh T. (2017). Efficacy and effectiveness of screen and treat policies in prevention of type 2 diabetes: systematic review and meta-analysis of screening tests and interventions. BMJ (Clinical research ed).

[16] Shahim B., De Bacquer D., De Backer G., Gyberg V., Kotseva K., Mellbin L. (2017). The Prognostic Value of Fasting Plasma Glucose, Two-Hour Postload Glucose, and HbA(1c) in Patients With Coronary Artery Disease: A Report From EUROASPIRE IV: A Survey From the European Society of Cardiology. Diabetes care.

[17] (2001). Glucose tolerance and cardiovascular mortality: comparison of fasting and 2-hour diagnostic criteria. Archives of internal medicine.

[18] Ke C., Narayan K.M.V., Chan J.C.N., Jha P., Shah B.R. (2022). Pathophysiology, phenotypes and management of type 2 diabetes mellitus in Indian and Chinese populations. Nature reviews Endocrinology.

[19] Zhang M., Wang L., Wu J., Huang Z., Zhao Z., Zhang X. (2022). Data Resource Profile: China Chronic Disease and Risk Factor Surveillance (CCDRFS). Int J Epidemiol.

[20] Liu Y., Wang L., Pang R., Mo N., Hu Y., Deng Q. (2015). Designing and implementation of a web-based quality monitoring system for plasma glucose measurement in multicenter population study.

[21] ElSayed N.A., Aleppo G., Aroda V.R., Bannuru R.R., Brown F.M., Bruemmer D. (2023). 2. Classification and Diagnosis of Diabetes: Standards of Care in Diabetes-2023. Diabetes care.

[22] Yang G., Rao C., Ma J., Wang L., Wan X., Dubrovsky G. (2006). Validation of verbal autopsy procedures for adult deaths in China. International journal of epidemiology.

[23] Poon A.K., Meyer M.L., Reaven G., Knowles J.W., Selvin E., Pankow J.S. (2018). Short-Term Repeatability of Insulin Resistance Indexes in Older Adults: The Atherosclerosis Risk in Communities Study. J Clin Endocrinol Metab.

[24] Rutter C.E., Millard L.A.C., Borges M.C., Lawlor D.A. (2023). Exploring regression dilution bias using repeat measurements of 2858 variables in ≤49 000 UK Biobank participants. Int J Epidemiol.

[25] Selvin E., Crainiceanu C.M., Brancati F.L., Coresh J. (2007). Short-term variability in measures of glycemia and implications for the classification of diabetes. Arch Intern Med.

[26] Auger N., Feuillet P., Martel S., Lo E., Barry A.D., Harper S. (2014). Mortality inequality in populations with equal life expectancy: Arriaga's decomposition method in SAS, Stata, and Excel. Annals of epidemiology.

[27] Menke A., Casagrande S., Cowie C.C. (2018). Contributions of A1c, fasting plasma glucose, and 2-hour plasma glucose to prediabetes prevalence: NHANES 2011-2014. Annals of epidemiology.

[28] Olson D.E., Rhee M.K., Herrick K., Ziemer D.C., Twombly J.G., Phillips L.S. (2010). Screening for diabetes and pre-diabetes with proposed A1C-based diagnostic criteria. Diabetes care.

[29] Schlesinger S., Neuenschwander M., Barbaresko J., Lang A., Maalmi H., Rathmann W. (2022). Prediabetes and risk of mortality, diabetes-related complications and comorbidities: umbrella review of meta-analyses of prospective studies. Diabetologia.

[30] Warren B., Pankow J.S., Matsushita K., Punjabi N.M., Daya N.R., Grams M. (2017). Comparative prognostic performance of definitions of prediabetes: a prospective cohort analysis of the Atherosclerosis Risk in Communities (ARIC) study. Lancet Diabetes Endocrinol.

[31] Faerch K., Witte D.R., Tabak A.G., Perreault L., Herder C., Brunner E.J. (2013). Trajectories of cardiometabolic risk factors before diagnosis of three subtypes of type 2 diabetes: a post-hoc analysis of the longitudinal Whitehall II cohort study. Lancet Diabetes Endocrinol.

[32] Ferrannini G., Tuomilehto J., De Backer G., Kotseva K., Mellbin L., Schnell O. (2024). Dysglycaemia screening and its prognostic impact in patients with coronary artery disease: experiences from the EUROASPIRE IV and V cohort studies. Lancet Diabetes Endocrinol.

[33] Meijnikman A.S., De Block C.E.M., Dirinck E., Verrijken A., Mertens I., Corthouts B. (2017). Not performing an OGTT results in significant underdiagnosis of (pre)diabetes in a high risk adult Caucasian population. Int J Obes (Lond).

[34] Rooney M.R., Wallace A.S., Echouffo Tcheugui J.B., Fang M., Hu J., Lutsey P.L. (2025). Prediabetes is associated with elevated risk of clinical outcomes even without progression to diabetes. Diabetologia.

[35] Forouhi N.G., Balkau B., Borch-Johnsen K., Dekker J., Glumer C., Qiao Q. (2006). The threshold for diagnosing impaired fasting glucose: a position statement by the European Diabetes Epidemiology Group. Diabetologia.

[36] Sadiya A., Jakapure V., Kumar V. (2023). Ethnic Variability in Glucose and Insulin Response to Rice Among Healthy Overweight Adults: A Randomized Cross-Over Study. Diabetes, metabolic syndrome and obesity: targets and therapy.

[37] Whincup P.H., Gilg J.A., Owen C.G., Odoki K., Alberti K.G., Cook D.G. (2005). British South Asians aged 13-16 years have higher fasting glucose and insulin levels than Europeans. Diabetic medicine: a journal of the British Diabetic Association.

[38] Qiao Q., Dekker J.M., de Vegt F., Nijpels G., Nissinen A., Stehouwer C.D. (2004). Two prospective studies found that elevated 2-hr glucose predicted male mortality independent of fasting glucose and HbA1c. Journal of clinical epidemiology.

[39] Thanopoulou A., Karamanos B., Archimandritis A. (2010). Glycated hemoglobin, diabetes, and cardiovascular risk in nondiabetic adults. The New England journal of medicine.

[40] Kong X., Wang W. (2025). Prediabetes Phenotypes and All-Cause or Cardiovascular Mortality: Evidence From a Population-Based Study. Endocrine practice: official journal of the American College of Endocrinology and the American Association of Clinical Endocrinologists.

[41] Yang W., Lu J., Weng J., Jia W., Ji L., Xiao J. (2010). Prevalence of diabetes among men and women in China. N Engl J Med.

[42] Hsu W.C., Boyko E.J., Fujimoto W.Y., Kanaya A., Karmally W., Karter A. (2012). Pathophysiologic differences among Asians, native Hawaiians, and other Pacific Islanders and treatment implications. Diabetes Care.

[43] Kanaya A.M., Herrington D., Vittinghoff E., Ewing S.K., Liu K., Blaha M.J. (2014). Understanding the high prevalence of diabetes in U.S. south Asians compared with four racial/ethnic groups: the MASALA and MESA studies. Diabetes Care.

[44] Nathan D.M., Davidson M.B., DeFronzo R.A., Heine R.J., Henry R.R., Pratley R. (2007). Impaired fasting glucose and impaired glucose tolerance: implications for care. Diabetes Care.

[45] Martin S., Samuel M., Stow D., Ridsdale A.M., Chen J., Young K.G. (2025). Undiagnosed G6PD Deficiency in Black and Asian Individuals Is Prevalent and Contributes to Health Inequalities in Type 2 Diabetes Diagnosis and Complications. Diabetes care.

[46] Lu J., He J., Li M., Tang X., Hu R., Shi L. (2019). Predictive Value of Fasting Glucose, Postload Glucose, and Hemoglobin A(1c) on Risk of Diabetes and Complications in Chinese Adults. Diabetes care.

[47] Harding J.L., Soderberg S., Shaw J.E., Zimmet P.Z., Pauvaday V., Kowlessur S. (2012). All-cause cancer mortality over 15 years in multi-ethnic Mauritius: the impact of diabetes and intermediate forms of glucose tolerance. International journal of cancer.

[48] Zhou X.H., Qiao Q., Zethelius B., Pyörälä K., Söderberg S., Pajak A. (2010). Diabetes, prediabetes and cancer mortality. Diabetologia.

[49] Rao Kondapally Seshasai S., Kaptoge S., Thompson A., Di Angelantonio E., Gao P., Sarwar N. (2011). Diabetes mellitus, fasting glucose, and risk of cause-specific death. The New England journal of medicine.

[50] Bragg F., Holmes M.V., Iona A., Guo Y., Du H., Chen Y. (2017). Association Between Diabetes and Cause-Specific Mortality in Rural and Urban Areas of China. Jama.

[51] Nanayakkara N., Curtis A.J., Heritier S., Gadowski A.M., Pavkov M.E., Kenealy T. (2021). Impact of age at type 2 diabetes mellitus diagnosis on mortality and vascular complications: systematic review and meta-analyses. Diabetologia.

[bib52] 52Life expectancy associated with different ages at diagnosis of type 2 diabetes in high-income countries: 23 million

