# Supplementary material for: Associations of Different Definitions of Prediabetes and Diabetes with All-Cause and Cause-Specific Mortality: A Nationally Representative Cohort Study
Source: Mil Med Res. 2026 Apr 22;13(1):100028. doi: 10.1016/j.mmr.2026.100028 (PMC13127155; doi:10.1016/j.mmr.2026.100028)
Supplement: Supplementary file 1 — Supplementary material [file mmc1.docx]

**Supplementary Materials**

**Supplementary Methods**

Data Collection and Assessment of Covariates

HbA1c assay method and quality control

Regression dilution correction

Life expectancy

**Table S1.** Baseline characteristics of prediabetes by different definitions.

**Table S2.** Baseline characteristics of newly diagnosed diabetes by different definitions

**Table S3.** Number of deaths, cardiovascular mortality rates, adjusted hazard ratio by different definitions of prediabetes and newly diagnosed diabetes

**Table S4.** Number of deaths, cancer mortality rates, adjusted hazard ratio by different definitions of prediabetes and newly diagnosed diabetes

**Table S5.** Associations of different definitions of prediabetes and diabetes with cause-specific mortality: Fine–Gray competing-risks analyses.

**Table S6.** Association between different definitions of prediabetes and diabetes with all-cause and cause-specific mortality accounted for the complex survey design.

**Table S7.** Sensitivity analysis of associations after correction for regression dilution bias.

**Table S8.** Adjusted hazard ratio (95% CI) for all-cause mortality by different definitions of prediabetes and newly diagnosed diabetes and age

**Table S9.** Adjusted hazard ratio (95%CI) for all-cause mortality by different clinical categories of prediabetes and newly diagnosed diabetes and sex

**Table S10.** Adjusted hazard ratio (95% CI) for all-cause mortality by different definitions of prediabetes and newly diagnosed diabetes and residence

**Table S11.** Adjusted hazard ratio (95%CI) for all-cause mortality by different definitions of prediabetes and newly diagnosed diabetes and hypertension

**Figure S1.** Prevalence of prediabetes and newly diagnosed diabetes in the Chinese population aged ≥18 years by different definition and gender/age in 2013

**Figure S2.** Dose-response relationship between different definitions of prediabetes and all-cause and cause-specific mortality.

**Figure S3.** Dose-response relationship between different glycemic indicators and all-cause and cause-specific mortality, excluding deaths occurring within the first 3 years of follow-up and participants with major comorbidities at baseline.

**Figure S4.** Estimated years of life lost attributable to increased deaths from cardiovascular disease, cancer, and other causes in people with newly diagnosed diabetes by different definitions.

**Figure S5.** Sensitivity analysis of years of life lost due to cause-specific mortality using age-restricted life tables and competing-risk models.

**Supplementary Methods**

**Data Collection and Assessment of Covariates**

The survey included a comprehensive questionnaire administered by trained interviewers, anthropometric measurements, and blood sample collection and measurement. The questionnaire included demographic characteristics, socioeconomic backgrounds, lifestyle risk factors and medical histories. Alcohol consumption was classified as excessive (≥14 g/day for men and ≥8 g/day for women), non-drinker/moderate (0-14 g/day for men or 0-8 g/day for women). The Global Physical Activity Questionnaire was used to assess physical activity. Physical activity was categorized as inactive group (<150 min/week of exercise), or active group (≥150 min/week of exercise). Body weight and height were measured according to a standard protocol. Body mass index (BMI) was calculated from weight/height2 (kg/m2). According to the previous guidelines ^1^, low fruit and vegetable intake was defined as consuming less than 400 g of fruit and vegetable per day; and high red meat intake was defined as consuming more than 100 g of pork, beef and lamb per day. Hypertension was defined as systolic blood pressure ≥140 mmHg, or diastolic blood pressure ≥90 mmHg, or self-reported physician’s diagnosis, or taking anti-hypertensive medication for the past 2 weeks. Dyslipidemia was defined as total cholesterol≥6.2 mmol/L, or low density lipoprotein (LDL-C)≥4.1 mmol/L, or high density lipoprotein (HDL-C)<1.0 mmol/L, or triglyceride ≥2.3 mmol/L, or current use of lipid-lowering medications.

**References**

1. WHO. Global action plan for the prevention and control of noncommunicable diseases 2013–2020. World Health Organization. 2013.

**HbA1c assay method and quality control**

All HbA1c samples were shipped to a central certified laboratory (KingMed Diagnostics, Guangzhou, China) and measured within 1 month of collection, which is within the stability range recommended by the manufacturer. HbA1c was directly measured using quantitative high-performance liquid chromatography with a boronate affinity method (Bio-Rad D-10 Hemoglobin Analyzer) ^1,2^. This method is certified by the National Glycohemoglobin Standardization Program (NGSP) as traceable to the Diabetes Control and Complications Trial (DCCT) reference and regularly passes proficiency testing by the College of American Pathologists (CAP) ^1,2^.

The analyzer’s performance was regularly verified using daily maintenance, calibration, and quality control (QC) samples before and after each batch (~100–150 samples/day). Out-of-control (OOC) conditions were flagged by standard QC rules (1–3s or 2–2s). The coefficients of variation (CVs) were 1.16% and 1.22% at HbA1c levels of 5.7% and 9.4%, respectively. Four trained technicians with ≥4 years of experience conducted all measurements, and were blinded to participant clinical data, including diabetes status.

**References**

1.CLSI/NCCLS C24-A3. Statistical Quality Control for Quantitative Measurements: Principles and Definitions: Approved Guideline, 3rd edition. Wayne, PA, USA: Clinical and Laboratory Standards Institute, 2006.

2.NCCLS. Evaluation of Precision Performance of Quantitative Measurement Methods; Approved Guideline – Second Edition. NCCLS document EP5-A2 [ISBN 1-56238-542-9]. NCCLS, Wayne, Pennsylvania USA, 2004.

**Regression dilution correction**

To assess the potential impact of random measurement error in baseline glycaemic measures, we conducted sensitivity analyses addressing regression dilution bias ^1^. Regression dilution may arise when a single baseline measurement does not accurately represent an individual’s long-term exposure level, leading to attenuation of effect estimates toward the null ^2^. As repeat glycaemic measurements were not available in the present study, we used previously published reliability estimates as proxies for the regression dilution ratio, including an intraclass correlation coefficient (ICC) of 0.70 for FPG (The Atherosclerosis Risk in Communities Study) ^3^, an ICC of 0.75 for HbA1c (UK Biobank) ^4^, and a repeat-measure correlation coefficient of 0.89 for 2-hour OGTT glucose (NHANES III) ^5^. Regression dilution was addressed by rescaling the estimated log hazard ratios using these reliability coefficients (β_corrected = β_observed / ICC). The corrected results were compared with the primary findings to determine whether random measurement error could materially influence the observed associations.

**References**

1.Knuiman MW, Divitini ML, Buzas JS, Fitzgerald PE. Adjustment for regression dilution in epidemiological regression analyses. Ann Epidemiol. 1998;8(1):56-63. doi: 10.1016/s1047-2797(97)00107-5.

2.Hutcheon JA, Chiolero A, Hanley JA. Random measurement error and regression dilution bias. BMJ. 2010;340:c2289. doi: 10.1136/bmj.c2289.

3.Poon AK, Meyer ML, Reaven G, Knowles JW, Selvin E, Pankow JS, Couper D, Loehr L, Heiss G. Short-Term Repeatability of Insulin Resistance Indexes in Older Adults: The Atherosclerosis Risk in Communities Study. J Clin Endocrinol Metab. 2018;103(6):2175-81. doi: 10.1210/jc.2017-02437.

4.Rutter CE, Millard LAC, Borges MC, Lawlor DA. Exploring regression dilution bias using repeat measurements of 2858 variables in ≤49 000 UK Biobank participants. Int J Epidemiol. 2023;52(5):1545-56. doi: 10.1093/ije/dyad082.

5.Selvin E, Crainiceanu CM, Brancati FL, Coresh J. Short-term variability in measures of glycemia and implications for the classification of diabetes. Arch Intern Med. 2007;167(14):1545-51. doi: 10.1001/archinte.167.14.1545.

**Life expectancy**

We combined three pieces of information to estimate the life expectancy according to different definitions of prediabetes and newly diagnosed diabetes (henceforth “exposure groups”).

1. sex- and age-specific population of all-causes mortality rate in 2019 from the GBD website;
2. HRs of mortality in each exposure group versus the reference from China Chronic Disease and Risk Factors Surveillance;
3. sex- and age-specific population prevalence of each exposure group from the China Chronic Disease and Risk Factors Surveillance.

The lifetables for each of the exposure groups in male and female, separately, were built on the estimated population mortality rates in each exposure group. We estimated reductions in life-expectancy as differences in expectation of life at any given age between any two lifetables compared.

Population-based prevalence of exposure groups by sex and 5-year age intervals came from the China Chronic Disease and Risk Factors Surveillance. The sex- and age-specific prevalences of exposure groups were assumed to be constant in each 5-year interval until 90 years. For the population aged >90 years, the prevalences were assumed to be the same as that in the 86-90 years age group due to insufficient numbers of participants aged >90 years in dataset.

Population all-cause mortality rates per 100,000 were obtained in 5-year age-groups for the Chinese population during year 2019 from the GBD website (http://ghdx.healthdata.org/gbd-results-tool). The sex- and age-specific population mortality rates were assumed to be constant in each 5-year interval.

HRs for mortality associated with diabetes and prediabetes, assumed to be constant across age groups, were estimated from the China Chronic Disease and Risk Factors Surveillance. We fitted Cox proportional hazards regression models, with adjustment for age, education level, household income, smoking, alcohol consumption, physical activity, BMI, red meat intake, vegetable and fruit intakes, hypertension, dyslipidemia, and self-reported CVD and cancer.

We used an algebraic transformation to infer the age-specific mortality rates appropriate for the reference group *𝐼𝑅_𝑎0_* by sex as: ^1^

$${IR}_{a0} =\frac{{IR}_{a}}{P_{a0} +\sum_{j=1}^{2} P_{aj}\times{HR}_{j}}$$

Where *IR_a_* is the population mortality rate for age group a; *P_aj_* is the age-specific prevalence of exposure groups; *HR_j_* is the hazard ratio for comparison of group j versus reference group (j = 0). The age-specific mortality rates for each non-reference group were then inferred by multiplying the age-specific mortality rate for the reference group *𝐼𝑅_𝑎0_* and the *HR_j_*.

Finally, based on the sex- and age-specific mortality rate, life tables were built. Survival probability was set as 1 at the age of 40. The probability of surviving between ages x and x+1 was then estimated based on the probability of dying (mortality rate) between ages x and x+1, assuming that the survivor function declines linearly between ages x and x+1 ^2,3^. For the last open-ended age interval, the probability of surviving was set to 0. The life expectancy at any given age was derived by dividing the total person-years that would be lived beyond age x by the number of persons who survived to that age interval ^2^.

**References**

1.Woloshin S, Schwartz LM, Welch HG. The risk of death by age, sex, and smoking status in the United States: putting health risks in context. Journal of the National Cancer Institute. 2008;100(12):845-853.

2.Chiang CL. Life table and mortality analysis. 1978.

3.Elizabeth Arias, Xu J. United States life tables, 2019. 2022

**Table S1.** Baseline characteristics of prediabetes by different definitions.

|  | **ADA FPG 5.6-6.9 mmol/L** | **ADA HbA1c 5.7-6.4%** | **ADA or WHO 2hPG 7.8-11.0 mmol/L** | **WHO FPG 6.1-6.9 mmol/L** | **WHO/IEC HbA1c 6.0-6.4%** |
| --- | --- | --- | --- | --- | --- |
| **Age (years), mean ± SD** | 53.46±13.35 | 56.81±12.56 | 56.30±13.39 | 54.68±12.99 | 57.79±12.37 |
| **Male, %** | 44.06 | 39.91 | 41.78 | 45.5 | 39.24 |
| **Urban, %** | 44.24 | 45.24 | 46.14 | 44.51 | 47.61 |
| **Education (collage or above), %** | 4.98 | 3.98 | 4.57 | 4.63 | 4.03 |
| **Household income (≥20,000 yuan/year), %** | 54.48 | 50.32 | 51.31 | 53.35 | 49.64 |
| **Fasting plasma glucose (mmol/L), mean ± SD** | 6.04±0.35 | 5.77±0.93 | 5.90±0.93 | 6.43±0.25 | 6.04±1.03 |
| **HbA1c (%), mean ± SD** | 5.42±0.48 | 5.89±0.19 | 5.51±0.52 | 5.52±0.52 | 6.13±0.13 |
| **2-hour glucose (mmol/L), mean ± SD** | 7.06±2.18 | 7.31±2.60 | 8.95±0.89 | 7.682.47 | 8.15±3.01 |
| **BMI (≥24 kg/m^2^), %** | 58.79 | 58.63 | 59.34 | 59.26 | 63.69 |
| **Active smoker, %** | 24.88 | 24.1 | 23.19 | 25.85 | 23.04 |
| **Excessive alcohol drinker, %** | 14.64 | 11.53 | 14.12 | 16.34 | 11.35 |
| **<400 g/day of fruits or vegetables, %** | 55.46 | 55.43 | 55.32 | 54.95 | 54.81 |
| **≥100 g/day of red meat, %** | 32.13 | 31.69 | 31.01 | 32.06 | 30.95 |
| **Physical activity (≥150 min/week), %** | 91.08 | 90.48 | 89.6 | 91.09 | 90.37 |
| **Hypertension, %** | 41.63 | 45.85 | 49.05 | 47.11 | 51.18 |
| **Dyslipidemia, %** | 33.83 | 38.53 | 38.25 | 37.73 | 43.98 |
| **Self-reported CVD, %** | 2.86 | 3.55 | 3.79 | 3.28 | 4.11 |

**Table S2.** Baseline characteristics of newly diagnosed diabetes by different definitions

|  | **FPG, ≥7.0 mmol/L** | **HbA1c, ≥6.5%** | **2hPG, ≥11.1 mmol/L** |
| --- | --- | --- | --- |
| **Age (years), mean ± SD** | 55.12±12.56 | 56.11±12.20 | 58.03±12.73 |
| **Male, %** | 48.87 | 44.10 | 46.27 |
| **Urban, %** | 46.03 | 50.55 | 50.44 |
| **Education (collage or above), %** | 4.98 | 5.12 | 4.84 |
| **Household income (≥20,000 yuan/year), %** | 54.95 | 53.49 | 52.35 |
| **Fasting plasma glucose (mmol/L), mean ± SD** | 8.73±2.46 | 8.80±3.29 | 7.79±2.88 |
| **HbA1c (%), mean ± SD** | 6.58±1.64 | 7.76±1.55 | 6.63±1.62 |
| **2-hour glucose (mmol/L), mean ± SD** | 12.14±5.73 | 14.45±6.13 | 14.96±4.11 |
| **BMI (≥24 kg/m^2^), %** | 69.19 | 75.65 | 68.50 |
| **Active smoker, %** | 27.05 | 24.28 | 25.24 |
| **Excessive alcohol drinker, %** | 17.94 | 13.26 | 16.56 |
| **<400 g/day of fruits or vegetables, %** | 54.54 | 54.82 | 55.13 |
| **≥100 g/day of red meat, %** | 31.73 | 32.27 | 31.14 |
| **Physical activity (≥150 min/week), %** | 90.17 | 89.50 | 89.37 |
| **Hypertension, %** | 54.55 | 58.09 | 60.94 |
| **Dyslipidemia, %** | 49.45 | 57.41 | 50.16 |
| **Self-reported CVD, %** | 3.42 | 4.12 | 4.74 |

**Table S3.** Number of deaths, cardiovascular mortality rates, adjusted hazard ratio by different definitions of prediabetes and newly diagnosed diabetes

|  |  | **Deaths number** | **Death rate (per 100,000 person years)** | **HR (95% CI)** | **HR (95% CI) further adjusted for FPG** | **HR (95% CI) further adjusted for HbA1c** | **HR (95% CI) further adjusted for 2hPG** | **HR (95% CI) further adjusted for other two definitions** |
| --- | --- | --- | --- | --- | --- | --- | --- | --- |
| **ADA FPG definition** | **<5.6 mmol/L** | 1337 | 167.71 | Ref. | N/A | Ref. | Ref. |  |
|  | **5.6-6.9 mmol/L** | 813 | 206.22 | 0.97 (0.89, 1.06) |  | 0.96 (0.87, 1.04) | 0.93 (0.85, 1.02) | 0.93 (0.85, 1.01) |
|  | **≥7.0 mmol/L** | 177 | 288.21 | 1.25 (1.07, 1.47) |  | 1.07 (0.89, 1.28) | 0.97 (0.80, 1.17) | 0.93 (0.76, 1.14) |
|  | **Per one SD increment in FPG** | - | - | 1.05 (1.02, 1.10) |  | 1.00 (0.95, 1.05) | 0.98 (0.93, 1.03) | 0.95 (0.90, 1.01) |
| **ADA HbA1c definition** | **<5.7%** | 1599 | 160.37 | Ref. | Ref. | N/A | Ref. | Ref. |
|  | **5.7-6.4%** | 617 | 278.84 | 1.12 (1.02, 1.23) | 1.11 (1.01, 1.23) |  | 1.08 (0.98, 1.19) | 1.09 (0.99, 1.20) |
|  | **≥6.5%** | 111 | 321.56 | 1.37 (1.13, 1.67) | 1.28 (1.02, 1.61) |  | 1.02 (0.80, 1.29) | 1.05 (0.83, 1.35) |
|  | **Per one SD increment in HbA1c** |  |  | 1.10 (1.06, 1.14) | 1.10 (1.05, 1.15) |  | 1.04 (0.99, 1.09) | 1.06 (1.01, 1.12) |
| **ADA or WHO 2hPG definition** | **<7.8 mmol/L** | 1577 | 154.15 | Ref. | Ref. | Ref. | N/A | Ref. |
|  | **7.8-11.0 mmol/L** | 508 | 294.58 | 1.13 (1.02, 1.25) | 1.12 (1.01, 1.24) | 1.11 (1.00, 1.23) |  | 1.12 (1.01, 1.24) |
|  | **≥11.1 mmol/L** | 242 | 421.31 | 1.44 (1.25, 1.65) | 1.41 (1.21, 1.65) | 1.31 (1.12, 1.53) |  | 1.34 (1.14, 1.57) |
|  | **Per one SD increment in 2hPG** | - | - | 1.11 (1.07, 1.14) | 1.12 (1.07, 1.17) | 1.08 (1.04, 1.13) |  | 1.10 (1.05, 1.15) |
| **WHO FPG definition** | **<6.1 mmol/L** | 1811 | 173.04 | Ref. | N/A | Ref. | Ref. | Ref. |
|  | **6.1-6.9 mmol/L** | 339 | 233.98 | 1.02 (0.91, 1.15) |  | 1.00 (0.89, 1.12) | 0.96 (0.85, 1.08) | 0.96 (0.85, 1.08) |
|  | **≥7.0 mmol/L** | 177 | 288.21 | 1.27 (1.09, 1.48) |  | 1.09 (0.91, 1.31) | 1.00 (0.83, 1.21) | 0.96 (0.79, 1.17) |
|  | **Per one SD increment in**  **FPG** | - | - | 1.05 (1.02, 1.10) |  | 1.00 (0.95, 1.05) | 0.98 (0.93, 1.03) | 0.95 (0.90, 1.01) |
| **WHO/IEC HbA1c definition** | **<6.0%** | 1985 | 172.69 | Ref. | Ref. | N/A | Ref. | Ref. |
|  | **6.0-6.4%** | 231 | 335.14 | 1.25 (1.09, 1.43) | 1.23 (1.07, 1.42) |  | 1.18 (1.03, 1.36) | 1.19 (1.03, 1.37) |
|  | **≥6.5%** | 111 | 321.56 | 1.36 (1.12, 1.65) | 1.27 (1.02, 1.60) |  | 1.02 (0.81, 1.29) | 1.06 (0.83, 1.35) |
|  | **Per one SD increment in HbA1c** | - | - | 1.10 (1.06, 1.14) | 1.10 (1.05, 1.15) |  | 1.04 (0.99, 1.09) | 1.06 (1.01, 1.12) |

The HRs were adjusted for age (five-year group), sex (male, or female), residence (urban, or rural), education (junior high school and below, high school, college and above), household income (<¥20,000/year, ≥¥20,000/year, or not answer/don’t know), smoking (never smoked, past smoker, or active smoker), alcohol consumption (excessive, rare/nondrinker), physical activity (<150 min/week, or ≥150 min/week), red meat intakes (<100 g/day, or ≥100 g/day), vegetable and fruit intakes (<400 g/day, or ≥400 g/day), body mass index (<18.5, 18.5-23.9, 24-27.9, ≥28 kg/m^2^), hypertension (yes, or no), dyslipidemia (yes, or no) and self-reported CVD (yes, or no) and cancer (yes, or no). FPG, fasting plasma glucose; 2hPG, 2-hour postload glucose.

**Table S4.** Number of deaths, cancer mortality rates, adjusted hazard ratio by different definitions of prediabetes and newly diagnosed diabetes

|  |  | **Deaths number** | **Death rate (per 100,000 person years)** | **HR (95% CI)** | **HR (95% CI) further adjusted for FPG** | **HR (95% CI) further adjusted for HbA1c** | **HR (95% CI) further adjusted for 2hPG** | **HR (95% CI) further adjusted for other two definitions** |
| --- | --- | --- | --- | --- | --- | --- | --- | --- |
| **ADA FPG definition** | **<5.6 mmol/L** | 973 | 122.05 | Ref. | N/A | Ref. | Ref. |  |
|  | **5.6-6.9 mmol/L** | 586 | 148.64 | 1.01 (0.91, 1.12) |  | 1.00 (0.90, 1.11) | 0.95 (0.85, 1.05) | 0.95 (0.85, 1.04) |
|  | **≥7.0 mmol/L** | 114 | 185.63 | 1.15 (0.95, 1.40) |  | 1.06 (0.85, 1.32) | 0.81 (0.64, 1.03) | 0.83 (0.65, 1.06) |
|  | **Per one SD increment in FPG** | - | - | 1.06 (1.02, 1.11) |  | 1.05 (0.99, 1.11) | 0.97 (0.92, 1.03) | 0.98 (0.92, 1.05) |
| **ADA HbA1c definition** | **<5.7%** | 1203 | 120.65 | Ref. | Ref. | N/A | Ref. | Ref. |
|  | **5.7-6.4%** | 396 | 178.96 | 1.06 (0.94, 1.19) | 1.04 (0.93, 1.18) |  | 1.01 (0.90, 1.13) | 1.01 (0.90, 1.14) |
|  | **≥6.5%** | 74 | 214.38 | 1.36 (1.07, 1.72) | 1.20 (0.90, 1.59) |  | 0.90 (0.67, 1.21) | 0.94 (0.70, 1.27) |
|  | **Per one SD increment in HbA1c** |  |  | 1.05 (1.01, 1.10) | 1.02 (0.96, 1.08) |  | 0.97 (0.91, 1.02) | 0.97 (0.91, 1.03) |
| **ADA or WHO 2hPG definition** | **<7.8 mmol/L** | 1205 | 117.79 | Ref. | Ref. | Ref. | N/A | Ref. |
|  | **7.8-11.0 mmol/L** | 311 | 180.34 | 1.09 (0.96, 1.24) | 1.08 (0.95 1.23) | 1.09 (0.96, 1.24) |  | 1.08 (0.95, 1.23) |
|  | **≥11.1 mmol/L** | 157 | 273.33 | 1.49 (1.26, 1.77) | 1.44 (1.19, 1.75) | 1.48 (1.22, 1.78) |  | 1.45 (1.19, 1.76) |
|  | **Per one SD increment in 2hPG** | - | - | 1.12 (1.08, 1.17) | 1.14 (1.09, 1.20) | 1.15 (1.09, 1.20) |  | 1.15 (1.09, 1.21) |
| **WHO FPG definition** | **<6.1 mmol/L** | 1335 | 127.56 | Ref. | N/A | Ref. | Ref. | Ref. |
|  | **6.1-6.9 mmol/L** | 224 | 154.61 | 0.97 (0.85, 1.12) |  | 0.96 (0.83, 1.11) | 0.90 (0.78, 1.04) | 0.90 (0.78, 1.07) |
|  | **≥7.0 mmol/L** | 114 | 185.63 | 1.15 (0.94, 1.39) |  | 1.05 (0.85, 1.31) | 0.81 (0.64, 1.02) | 0.83 (0.66, 1.06) |
|  | **Per one SD increment in FPG** | - | - | 1.06 (1.02, 1.11) |  | 1.05 (0.99, 1.11) | 0.97 (0.92, 1.03) | 0.98 (0.92, 1.05) |
| **WHO/IEC HbA1c definition** | **<6.0%** | 1477 | 128.50 | Ref. | Ref. | N/A | Ref. | Ref. |
|  | **6.0-6.4%** | 122 | 177.00 | 0.98 (0.82, 1.19) | 0.96 (0.80, 1.16) |  | 0.91 (0.75, 1.20) | 0.91(0.75, 1.10) |
|  | **≥6.5%** | 74 | 214.38 | 1.33 (1.05, 1.69) | 1.17 (0.89, 1.55) |  | 0.88 (0.66, 1.17) | 0.91 (0.67, 1.2.) |
|  | **Per one SD increment in HbA1c** | - | - | 1.05 (1.01, 1.10) | 1.02 (0.96, 1.08) |  | 0.97 (0.91, 1.02) | 0.97 (0.91, 1.03) |

The HRs were adjusted for age (five-year group), sex (male, or female), residence (urban, or rural), education (junior high school and below, high school, college and above), household income (<¥20,000/year, ≥¥20,000/year, or not answer/don’t know), smoking (never smoked, past smoker, or active smoker), alcohol consumption (excessive, rare/nondrinker), physical activity (<150 min/week, or ≥150 min/week), red meat intakes (<100 g/day, or ≥100 g/day), vegetable and fruit intakes (<400 g/day, or ≥400 g/day), body mass index (<18.5, 18.5-23.9, 24-27.9, ≥28 kg/m^2^), hypertension (yes, or no), dyslipidemia (yes, or no) and self-reported CVD (yes, or no) and cancer (yes, or no). FPG, fasting plasma glucose; 2hPG, 2-hour postload glucose.

**Table S5.** Associations of different definitions of prediabetes and diabetes with cause-specific mortality: Fine–Gray competing-risks analyses.

|  |  | **HR (95% CI)** | | |
| --- | --- | --- | --- | --- |
|  |  | **CVD mortality** | **Cancer mortality** |  |
| **ADA FPG definition** | **<5.6 mmol/L** | Ref. | Ref. |  |
|  | **5.6-6.9 mmol/L** | 1.01 (0.93, 1.10) | 0.99 (0.90, 1.09) |  |
|  | **≥7.0 mmol/L** | 1.43 (1.25, 1.64) | 1.14 (0.95, 1.36) |  |
|  | **Per one SD increment in FPG** | 1.09 (1.06, 1.13) | 1.05 (1.01, 1.10) |  |
| **ADA HbA1c definition** | **<5.7%** | Ref. | Ref. |  |
|  | **5.7-6.4%** | 1.09 (1.00, 1.18) | 1.06 (0.95, 1.18) |  |
|  | **≥6.5%** | 1.51 (1.27, 1.79) | 1.29 (1.03, 1.62) |  |
|  | **Per one SD increment in HbA1c** | 1.11 (1.08, 1.15) | 1.04 (1.00, 1.10) |  |
| **ADA or WHO 2hPG definition** | **<7.8 mmol/L** | Ref. | Ref. |  |
|  | **7.8-11.0 mmol/L** | 1.13 (1.03, 1.24) | 1.09 (0.97, 1.23) |  |
|  | **≥11.1 mmol/L** | 1.56 (1.38, 1.77) | 1.45 (1.24, 1.70) |  |
|  | **Per one SD increment in 2hPG** | 1.12 (1.09, 1.15) | 1.11 (1.07, 1.15) |  |
| **WHO FPG definition** | **<6.1 mmol/L** | Ref. | Ref. |  |
|  | **6.1-6.9 mmol/L** | 1.04 (0.94, 1.16) | 1.00 (0.88, 1.14) |  |
|  | **≥7.0 mmol/L** | 1.44 (1.25, 1.64) | 1.14 (0.95, 1.36) |  |
|  | **Per one SD increment in**  **FPG** | 1.09 (1.06, 1.13) | 1.05 (1.01, 1.10) |  |
| **WHO/IEC HbA1c definition** | **<6.0%** | Ref. | Ref. |  |
|  | **6.0-6.4%** | 1.21 (1.07, 1.37) | 1.02 (0.86, 1.21) |  |
|  | **≥6.5%** | 1.51 (1.27, 1.79) | 1.27 (1.02, 1.59) |  |
|  | **Per one SD increment in HbA1c** | 1.11 (1.08, 1.15) | 1.04 (1.00, 1.10) |  |

The HRs were adjusted for age (five-year group), sex (male, or female), residence (urban, or rural), education (junior high school and below, high school, college and above), household income (<¥20,000/year, ≥¥20,000/year, or not answer/don’t know), smoking (never smoked, past smoker, or active smoker), alcohol consumption (excessive, rare/nondrinker), physical activity (<150 min/week, or ≥150 min/week), red meat intakes (<100 g/day, or ≥100 g/day), vegetable and fruit intakes (<400 g/day, or ≥400 g/day), body mass index (<18.5, 18.5-23.9, 24-27.9, ≥28 kg/m^2^), hypertension (yes, or no), dyslipidemia (yes, or no) and self-reported CVD (yes, or no) and cancer (yes, or no). FPG, fasting plasma glucose; 2hPG, 2-hour postload glucose.

**Table S6.** Association between different definitions of prediabetes and diabetes with all-cause and cause-specific mortality accounted for the complex survey design.

|  |  | **HR (95% CI)** | | |
| --- | --- | --- | --- | --- |
|  |  | **All-cause mortality** | **CVD mortality** | **Cancer mortality** |
| **ADA FPG definition** | **<5.6 mmol/L** | Ref. | Ref. | Ref. |
|  | **5.6-6.9 mmol/L** | 0.99 (0.93, 1.05) | 0.97 (0.88, 1.08) | 1.00 (0.91, 1.11) |
|  | **≥7.0 mmol/L** | 1.25 (1.12, 1.39) | 1.25 (1.06, 1.47) | 1.15 (0.94, 1.42) |
|  | **Per one SD increment in FPG** | 1.06 (1.03, 1.09) | 1.05 (1.01, 1.10) | 1.06 (1.02, 1.11) |
| **ADA HbA1c definition** | **<5.7%** | Ref. | Ref. | Ref. |
|  | **5.7-6.4%** | 1.14 (1.07, 1.21) | 1.12 (1.02, 1.24) | 1.06 (0.93, 1.20) |
|  | **≥6.5%** | 1.40 (1.23, 1.59) | 1.37 (1.12, 1.68) | 1.36 (1.07, 1.73) |
|  | **Per one SD increment in HbA1c** | 1.08 (1.06, 1.11) | 1.10 (1.05, 1.14) | 1.05 (1.00, 1.11) |
| **ADA or WHO 2hPG definition** | **<7.8 mmol/L** | Ref. | Ref. | Ref. |
|  | **7.8-11.0 mmol/L** | 1.13 (1.06, 1.21) | 1.13 (1.01, 1.25) | 1.09 (0.96, 1.23) |
|  | **≥11.1 mmol/L** | 1.50 (1.36, 1.65) | 1.44 (1.25, 1.66) | 1.49 (1.25, 1.79) |
|  | **Per one SD increment in 2hPG** | 1.11 (1.09, 1.14) | 1.11 (1.07, 1.15) | 1.12 (1.08, 1.17) |
| **WHO FPG definition** | **<6.1 mmol/L** | Ref. | Ref. | Ref. |
|  | **6.1-6.9 mmol/L** | 1.02 (0.95, 1.09) | 1.02 (0.90, 1.15) | 0.97 (0.84, 1.13) |
|  | **≥7.0 mmol/L** | 1.26 (1.13, 1.40) | 1.27 (1.08, 1.49) | 1.08 (0.79, 1.46) |
|  | **Per one SD increment in**  **FPG** | 1.06 (1.03, 1.09) | 1.05 (1.01, 1.10) | 1.06 (1.02, 1.11) |
| **WHO/IEC HbA1c definition** | **<6.0%** | Ref. | Ref. | Ref. |
|  | **6.0-6.4%** | 1.23 (1.12, 1.34) | 1.25 (1.08, 1.44) | 0.98 (0.81, 1.19) |
|  | **≥6.5%** | 1.38 (1.22, 1.56) | 1.36 (1.11, 1.66) | 1.33 (1.05, 1.69) |
|  | **Per one SD increment in HbA1c** | 1.08 (1.06, 1.11) | 1.10 (1.05, 1.14) | 1.05 (1.00, 1.11) |

The HRs were estimated using survey-weighted Cox proportional hazards models accounting for the complex survey design, including sampling weights, stratification, and clustering. The HRs were adjusted for age (five-year group), sex (male, or female), residence (urban, or rural), education (junior high school and below, high school, college and above), household income (<¥20,000/year, ≥¥20,000/year, or not answer/don’t know), smoking (never smoked, past smoker, or active smoker), alcohol consumption (excessive, rare/nondrinker), physical activity (<150 min/week, or ≥150 min/week), red meat intakes (<100 g/day, or ≥100 g/day), vegetable and fruit intakes (<400 g/day, or ≥400 g/day), body mass index (<18.5, 18.5-23.9, 24-27.9, ≥28 kg/m^2^), dyslipidemia (yes, or no), hypertension (yes, or no), and self-reported CVD (yes, or no) and cancer (yes, or no).

**Table S7.** Sensitivity analysis of associations after correction for regression dilution bias.

|  |  | **HR (95% CI)** | | |
| --- | --- | --- | --- | --- |
|  |  | **All-cause mortality** | **CVD mortality** | **Cancer mortality** |
| **ADA FPG definition** | **<5.6 mmol/L** | 1 (ref.) | 1 (ref.) | 1 (ref.) |
|  | **5.6-6.9 mmol/L** | 0.98 (0.91, 1.05) | 0.96 (0.85, 1.09) | 1.01 (0.87, 1.17) |
|  | **≥7.0 mmol/L** | 1.37 (1.20, 1.57) | 1.37 (1.10, 1.73) | 1.23 (0.93, 1.62) |
|  | **Per one SD increment in FPG** | 1.08 (1.05, 1.12) | 1.08 (1.02, 1.14) | 1.09 (1.03, 1.16) |
| **ADA HbA1c definition** | **<5.7%** | 1 (ref.) | 1 (ref.) | 1 (ref.) |
|  | **5.7-6.4%** | 1.19 (1.10, 1.28) | 1.17 (1.03, 1.32) | 1.08 (0.92, 1.25) |
|  | **≥6.5%** | 1.56 (1.34, 1.83) | 1.52 (1.17, 1.98) | 1.50 (1.09, 2.07) |
|  | **Per one SD increment in HbA1c** | 1.11 (1.08, 1.15) | 1.13 (1.08, 1.19) | 1.07 (1.01, 1.14) |
| **ADA or WHO 2hPG definition** | **<7.8 mmol/L** | 1 (ref.) | 1 (ref.) | 1 (ref.) |
|  | **7.8-11.0 mmol/L** | 1.15 (1.07, 1.23) | 1.14 (1.02, 1.28) | 1.10 (0.95, 1.27) |
|  | **≥11.1 mmol/L** | 1.58 (1.44, 1.73) | 1.50 (1.29, 1.76) | 1.57 (1.30, 1.90) |
|  | **Per one SD increment in 2hPG** | 1.13 (1.11, 1.15) | 1.12 (1.08, 1.16) | 1.14 (1.09, 1.19) |
| **WHO FPG definition** | **<6.1 mmol/L** | 1 (ref.) | 1 (ref.) | 1 (ref.) |
|  | **6.1-6.9 mmol/L** | 1.03 (0.93, 1.13) | 1.03 (0.87, 1.22) | 0.96 (0.79, 1.18) |
|  | **≥7.0 mmol/L** | 1.39 (1.22, 1.59) | 1.40 (1.12, 1.76) | 1.21 (0.92, 1.60) |
|  | **Per one SD increment in**  **FPG** | 1.08 (1.05, 1.12) | 1.08 (1.02, 1.14) | 1.09 (1.03, 1.16) |
| **WHO/IEC HbA1c definition** | **<6.0%** | 1 (ref.) | 1 (ref.) | 1 (ref.) |
|  | **6.0-6.4%** | 1.31 (1.17, 1.46) | 1.34 (1.12, 1.62) | 0.98 (0.76, 1.26) |
|  | **≥6.5%** | 1.53 (1.31, 1.79) | 1.50 (1.16, 1.95) | 1.47 (1.07, 2.01) |
|  | **Per one SD increment in HbA1c** | 1.11 (1.08, 1.15) | 1.13 (1.08, 1.19) | 1.07 (1.01, 1.14) |

The HRs were adjusted for age (five-year group), sex (male, or female), residence (urban, or rural), education (junior high school and below, high school, college and above), household income (<¥20,000/year, ≥¥20,000/year, or not answer/don’t know), smoking (never smoked, past smoker, or active smoker), alcohol consumption (excessive, rare/nondrinker), physical activity (<150 min/week, or ≥150 min/week), red meat intakes (<100 g/day, or ≥100 g/day), vegetable and fruit intakes (<400 g/day, or ≥400 g/day), body mass index (<18.5, 18.5-23.9, 24-27.9, ≥28 kg/m^2^), hypertension (yes, or no), dyslipidemia (yes, or no) and self-reported CVD (yes, or no) and cancer (yes, or no). FPG, fasting plasma glucose; 2hPG, 2-hour postload glucose.

**Table S8.** Adjusted hazard ratio (95% CI) for all-cause mortality by different definitions of prediabetes and newly diagnosed diabetes and age

|  |  | **Age <65 years** | **Age ≥ 65 years** | ***P _interaction_*** |
| --- | --- | --- | --- | --- |
| **ADA FPG definition** | **<5.6 mmol/L** | Ref. | Ref. |  |
|  | **5.6-6.9 mmol/L** | 0.96 (0.89, 1.04) | 1.00 (0.94, 1.07) | 0.94 |
|  | **≥7.0 mmol/L** | 1.23 (1.06, 1.42) | 1.26 (1.11, 1.42) | 0.57 |
| **ADA HbA1c definition** | **<5.7%** | Ref. | Ref. |  |
|  | **5.7-6.4%** | 1.19 (1.09, 1.30) | 1.10 (1.02, 1.18) | 0.71 |
|  | **≥6.5%** | 1.46 (1.22, 1.75) | 1.35 (1.16, 1.58) | 0.93 |
| **ADA or WHO 2hPG definition** | **<7.8 mmol/L** | Ref. | Ref. |  |
|  | **7.8-11.0 mmol/L** | 1.27 (1.15, 1.40) | 1.05 (0.97, 1.13) | 0.03 |
|  | **≥11.1 mmol/L** | 1.75 (1.53, 2.00) | 1.37 (1.23, 1.52) | 0.02 |
| **WHO FPG definition** | **<6.1 mmol/L** | Ref. | Ref. |  |
|  | **6.1-6.9 mmol/L** | 1.05 (0.94, 1.16) | 0.99 (0.91, 1.09) | 0.74 |
|  | **≥7.0 mmol/L** | 1.26 (1.09, 1.45) | 1.25 (1.11, 1.41) | 0.78 |
| **WHO/IEC HbA1c definition** | **<6.0%** | Ref. | Ref. |  |
|  | **6.0-6.4%** | 1.36 (1.19, 1.56) | 1.15 (1.04, 1.28) | 0.19 |
|  | **≥6.5%** | 1.44 (1.21, 1.72) | 1.34 (1.15, 1.55) | 0.92 |

The HRs were adjusted for age (five-year group), sex (male, or female), residence (urban, or rural), education (junior high school and below, high school, college and above), household income (<¥20,000/year, ≥¥20,000/year, or not answer/don’t know), smoking (never smoked, past smoker, or active smoker), alcohol consumption (excessive, rare/nondrinker), physical activity (<150 min/week, or ≥150 min/week), red meat intakes (<100 g/day, or ≥100 g/day), vegetable and fruit intakes (<400 g/day, or ≥400 g/day), body mass index (<18.5, 18.5-23.9, 24-27.9, ≥28 kg/m^2^), hypertension (yes, or no), dyslipidemia (yes, or no) and self-reported CVD (yes, or no) and cancer (yes, or no). FPG, fasting plasma glucose; 2hPG, 2-hour postload glucose.

**Table S9.** Adjusted hazard ratio (95%CI) for all-cause mortality by different clinical categories of prediabetes and newly diagnosed diabetes and sex

|  |  | **Males** | **Females** | ***P _interaction_*** |
| --- | --- | --- | --- | --- |
| **ADA FPG definition** | **<5.6 mmol/L** | Ref. | Ref. |  |
|  | **5.6-6.9 mmol/L** | 0.98 (0.92, 1.05) | 0.99 (0.91, 1.07) | 0.67 |
|  | **≥7.0 mmol/L** | 1.28 (1.13, 1.44) | 1.20 (1.03, 1.40) | 0.68 |
| **ADA HbA1c definition** | **<5.7%** | Ref. | Ref. |  |
|  | **5.7-6.4%** | 1.20 (1.11, 1.29) | 1.06 (0.97, 1.15) | 0.17 |
|  | **≥6.5%** | 1.50 (1.29, 1.75) | 1.25 (1.04, 1.50) | 0.21 |
| **ADA or WHO 2hPG definition** | **<7.8 mmol/L** | Ref. | Ref. |  |
|  | **7.8-11.0 mmol/L** | 1.13 (1.04, 1.22) | 1.13 (1.03, 1.23) | 0.37 |
|  | **≥11.1 mmol/L** | 1.59 (1.43, 1.76) | 1.37 (1.20, 1.56) | 0.26 |
| **WHO FPG definition** | **<6.1 mmol/L** | Ref. | Ref. |  |
|  | **6.1-6.9 mmol/L** | 1.01 (0.92, 1.11) | 1.02 (0.92, 1.14) | 0.66 |
|  | **≥7.0 mmol/L** | 1.29 (1.15, 1.45) | 1.21 (1.04, 1.41) | 0.62 |
| **WHO/IEC HbA1c definition** | **<6.0%** | Ref. | Ref. |  |
|  | **6.0-6.4%** | 1.31 (1.18, 1.46) | 1.12 (0.99, 1.27) | 0.20 |
|  | **≥6.5%** | 1.47 (1.27, 1.71) | 1.25 (1.04, 1.50) | 0.25 |

The HRs were adjusted for age (five-year group), residence (urban, or rural), education (junior high school and below, high school, college and above), household income (<¥20,000/year, ≥¥20,000/year, or not answer/don’t know), smoking (never smoked, past smoker, or active smoker), alcohol consumption (excessive, rare/nondrinker), physical activity (<150 min/week, or ≥150 min/week), red meat intakes (<100 g/day, or ≥100 g/day), vegetable and fruit intakes (<400 g/day, or ≥400 g/day), body mass index (<18.5, 18.5-23.9, 24-27.9, ≥28 kg/m^2^), hypertension (yes, or no), dyslipidemia (yes, or no) and self-reported CVD (yes, or no) and cancer (yes, or no). FPG, fasting plasma glucose; 2hPG, 2-hour postload glucose.

**Table S10.** Adjusted hazard ratio (95% CI) for all-cause mortality by different definitions of prediabetes and newly diagnosed diabetes and residence

|  |  | **Urban areas** | **Rural areas** | ***P _interaction_*** |
| --- | --- | --- | --- | --- |
| **ADA FPG definition** | **<5.6 mmol/L** | Ref. | Ref. |  |
|  | **5.6-6.9 mmol/L** | 1.00 (0.92, 1.08) | 0.98 (0.92, 1.05) | 0.94 |
|  | **≥7.0 mmol/L** | 1.24 (1.08, 1.43) | 1.26 (1.11, 1.43) | 0.74 |
| **ADA HbA1c definition** | **<5.7%** | Ref. | Ref. |  |
|  | **5.7-6.4%** | 1.19 (1.09, 1.29) | 1.11 (1.03, 1.19) | 0.20 |
|  | **≥6.5%** | 1.30 (1.10, 1.54) | 1.50 (1.28, 1.76) | 0.14 |
| **ADA or WHO 2hPG definition** | **<7.8 mmol/L** | Ref. | Ref. |  |
|  | **7.8-11.0 mmol/L** | 1.15 (1.05, 1.26) | 1.12 (1.03, 1.21) | 0.50 |
|  | **≥11.1 mmol/L** | 1.44 (1.28, 1.63) | 1.56 (1.40, 1.75) | 0.37 |
| **WHO FPG definition** | **<6.1 mmol/L** | Ref. | Ref. |  |
|  | **6.1-6.9 mmol/L** | 1.10 (0.99, 1.22) | 0.97 (0.88, 1.06) | 0.09 |
|  | **≥7.0 mmol/L** | 1.26 (1.10, 1.45) | 1.26 (1.11, 1.42) | 0.89 |
| **WHO/IEC HbA1c definition** | **<6.0%** | Ref. | Ref. |  |
|  | **6.0-6.4%** | 1.17 (1.04, 1.33) | 1.28 (1.14, 1.43) | 0.32 |
|  | **≥6.5%** | 1.25 (1.06, 1.48) | 1.50 (1.28, 1.76) | 0.08 |

The HRs were adjusted for age (five-year group), sex (male, or female), education (junior high school and below, high school, college and above), household income (<¥20,000/year, ≥¥20,000/year, or not answer/don’t know), smoking (never smoked, past smoker, or active smoker), alcohol consumption (excessive, rare/nondrinker), physical activity (<150 min/week, or ≥150 min/week), red meat intakes (<100 g/day, or ≥100 g/day), vegetable and fruit intakes (<400 g/day, or ≥400 g/day), body mass index (<18.5, 18.5-23.9, 24-27.9, ≥28 kg/m^2^), hypertension (yes, or no), dyslipidemia (yes, or no) and self-reported CVD (yes, or no) and cancer (yes, or no). FPG, fasting plasma glucose; 2hPG, 2-hour postload glucose.

**Table S11.** Adjusted hazard ratio (95%CI) for all-cause mortality by different definitions of prediabetes and newly diagnosed diabetes and hypertension

|  |  | **With hypertension** | **Without hypertension** | ***P _interaction_*** |
| --- | --- | --- | --- | --- |
| **ADA FPG definition** | **<5.6 mmol/L** | Ref. | Ref. |  |
|  | **5.6-6.9 mmol/L** | 0.95 (0.89, 1.02) | 1.04 (0.96, 1.13) | 0.07 |
|  | **≥7.0 mmol/L** | 1.21 (1.08, 1.36) | 1.34 (1.14, 1.57) | 0.25 |
| **ADA HbA1c definition** | **<5.7%** | Ref. | Ref. |  |
|  | **5.7-6.4%** | 1.15 (1.07, 1.24) | 1.11 (1.02, 1.21) | 0.94 |
|  | **≥6.5%** | 1.35 (1.17, 1.56) | 1.56 (1.27, 1.91) | 0.17 |
| **ADA/WHO 2hPG definition** | **<7.8 mmol/L** | Ref. | Ref. |  |
|  | **7.8-11.0 mmol/L** | 1.10 (1.02, 1.18) | 1.18 (1.07, 1.30) | 0.09 |
|  | **≥11.1 mmol/L** | 1.46 (1.32, 1.61) | 1.63 (1.41, 1.89) | 0.11 |
| **WHO FPG definition** | **<6.1 mmol/L** | Ref. | Ref. |  |
|  | **6.1-6.9 mmol/L** | 1.02 (0.94, 1.11) | 1.04 (0.93, 1.17) | 0.66 |
|  | **≥7.0 mmol/L** | 1.24 (1.11, 1.39) | 1.32 (1.13, 1.55) | 0.46 |
| **IEC HbA1c definition** | **<6.0%** | Ref. | Ref. |  |
|  | **6.0-6.4%** | 1.20 (1.08, 1.33) | 1.27 (1.11, 1.45) | 0.32 |
|  | **≥6.5%** | 1.32 (1.15, 1.52) | 1.55 (1.27, 1.90) | 0.14 |

The HRs were adjusted for age (five-year group), sex (male, or female), residence (urban, or rural), education (junior high school and below, high school, college and above), household income (<¥20,000/year, ≥¥20,000/year, or not answer/don’t know), smoking (never smoked, past smoker, or active smoker), alcohol consumption (excessive, rare/nondrinker), physical activity (<150 min/week, or ≥150 min/week), red meat intakes (<100 g/day, or ≥100 g/day), vegetable and fruit intakes (<400 g/day, or ≥400 g/day), body mass index (<18.5, 18.5-23.9, 24-27.9, ≥28 kg/m^2^), dyslipidemia (yes, or no) and self-reported CVD (yes, or no) and cancer (yes, or no). FPG, fasting plasma glucose; 2hPG, 2-hour postload glucose.

**Figure S1.** Prevalence of prediabetes and newly diagnosed diabetes in the Chinese population aged ≥18 years by different definition and gender/age in 2013


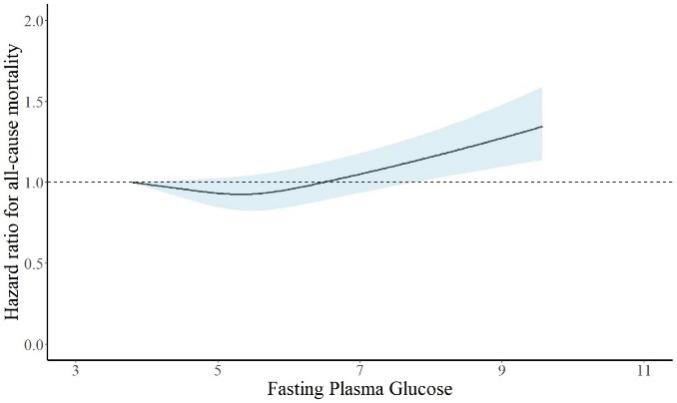

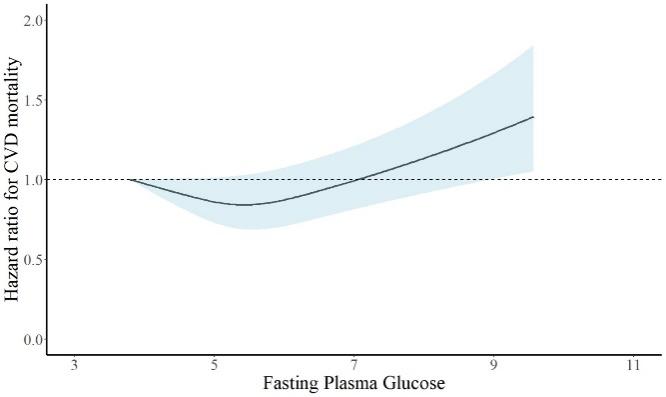

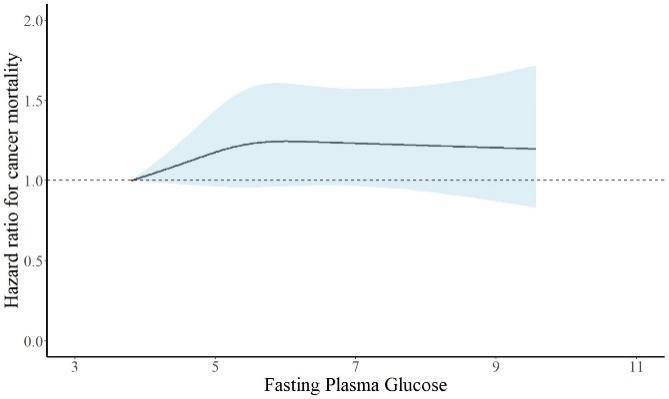


*P _overall_* =0·46

*P _nonlinear_* =0·34

*P _overall_* <0·001

*P _nonlinear_* =0·02

*P _overall_* <0·001

*P _nonlinear_* =0·03


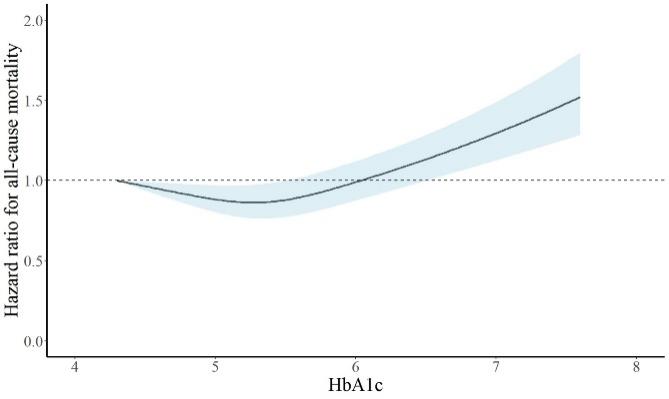

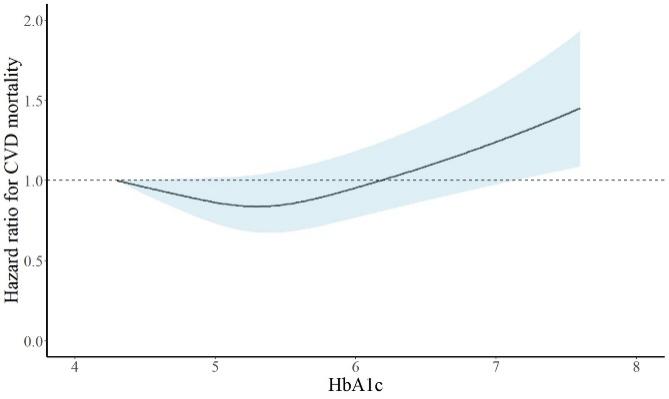

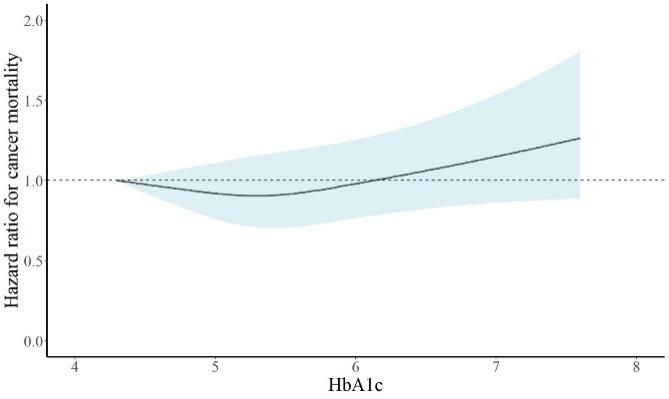


*P _overall_* <0·001

*P _nonlinear_* =0·04

*P _overall_* =0·45

*P _nonlinear_* =0·96

*P _overall_* <0·001

*P _nonlinear_* =0·009


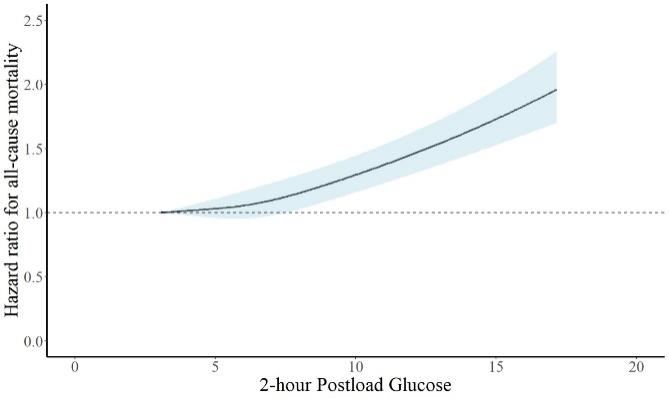

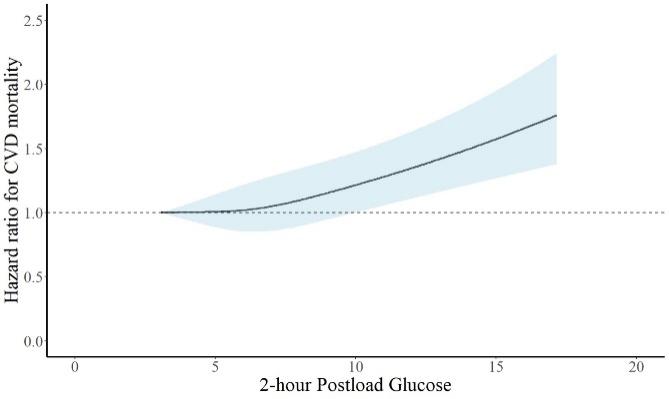

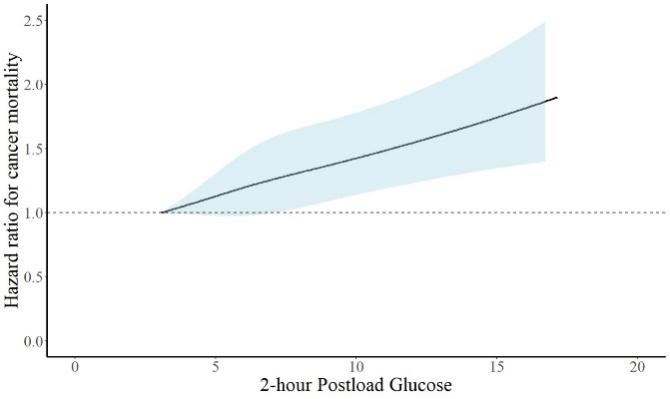


*P _overall_* <0·001

*P _nonlinear_* =0·24

*P _overall_* =<0·001

*P _nonlinear_* =0·27

*P _overall_* <0·001

*P _nonlinear_* =0·37

**Figure S2.** Dose-response relationship between different glycemic indicators and all-cause and cause-specific mortality.

The HRs were adjusted for age (five-year group), sex (male, or female), residence (urban, or rural), education (junior high school and below, high school, college and above), household income (<¥20,000/year, ≥¥20,000/year, or not answer/don’t know), smoking (never smoked, past smoker, or active smoker), alcohol consumption (excessive, rare/nondrinker), physical activity (<150 min/week, or ≥150 min/week), red meat intakes (<100 g/day, or ≥100 g/day), vegetable and fruit intakes (<400 g/day, or ≥400 g/day), body mass index (<18.5, 18.5-23.9, 24-27.9, ≥28 kg/m^2^), dyslipidemia (yes, or no), hypertension (yes, or no), and self-reported CVD (yes, or no) and cancer (yes, or no).


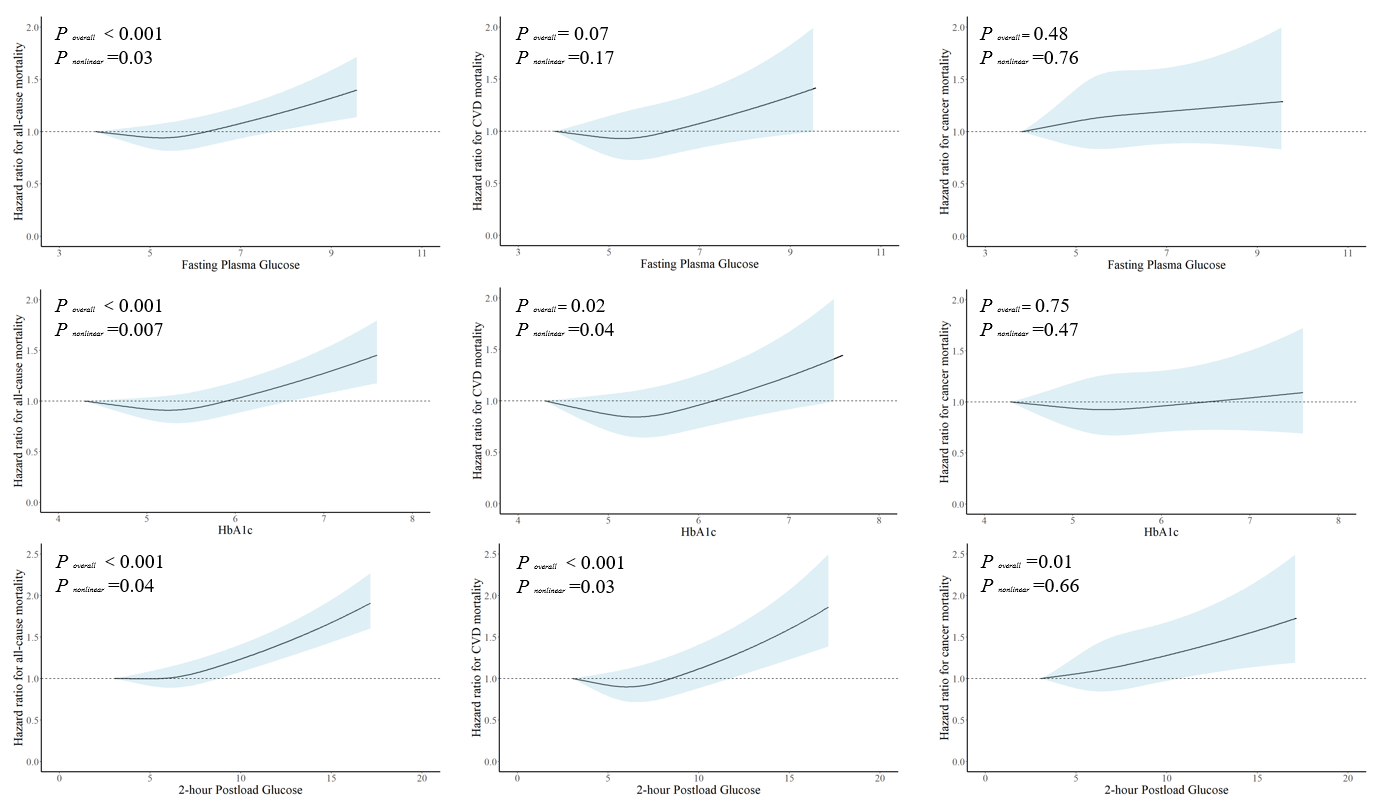


**Figure S3.** Dose-response relationship between different glycemic indicators and all-cause and cause-specific mortality, excluding deaths occurring within the first 3 years of follow-up and participants with major comorbidities at baseline.

The HRs were adjusted for age (five-year group), sex (male, or female), residence (urban, or rural), education (junior high school and below, high school, college and above), household income (<¥20,000/year, ≥¥20,000/year, or not answer/don’t know), smoking (never smoked, past smoker, or active smoker), alcohol consumption (excessive, rare/nondrinker), physical activity (<150 min/week, or ≥150 min/week), red meat intakes (<100 g/day, or ≥100 g/day), vegetable and fruit intakes (<400 g/day, or ≥400 g/day), body mass index (<18.5, 18.5-23.9, 24-27.9, ≥28 kg/m^2^), dyslipidemia (yes, or no), hypertension (yes, or no), and self-reported CVD (yes, or no) and cancer (yes, or no).


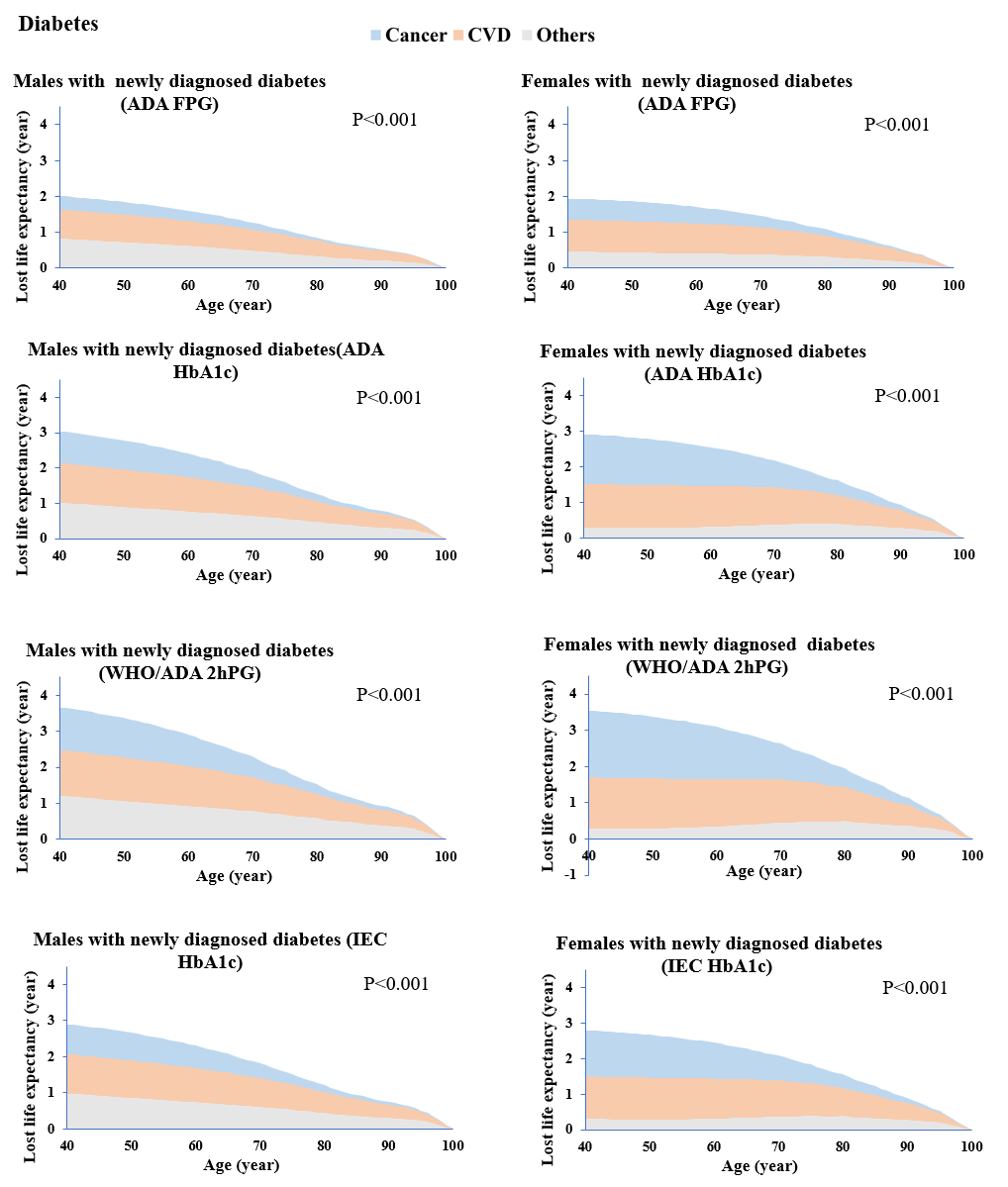


**Figure S4.** Estimated years of life lost attributable to increased deaths from cardiovascular disease, cancer, and other causes in people with newly diagnosed diabetes by different definition.

The HRs applied to estimate life expectancy were adjusted for age (five-year group), sex (male, or female), residence (urban, or rural), education (junior high school and below, high school, college and above), household income (<¥20,000/year, ≥¥20,000/year, or not answer/don’t know), smoking (never smoked, past smoker, or active smoker), alcohol consumption (excessive, rare/nondrinker), physical activity (<150 min/week, or ≥150 min/week), red meat intakes (<100 g/day, or ≥100 g/day), vegetable and fruit intakes (<400 g/day, or ≥400 g/day), body mass index (<18.5, 18.5-23.9, 24-27.9, ≥28 kg/m^2^), hypertension (yes, or no), dyslipidemia (yes, or no) and self-reported CVD (yes, or no) and cancer (yes, or no). FPG, fasting plasma glucose; 2hPG, 2-hour postload glucose.


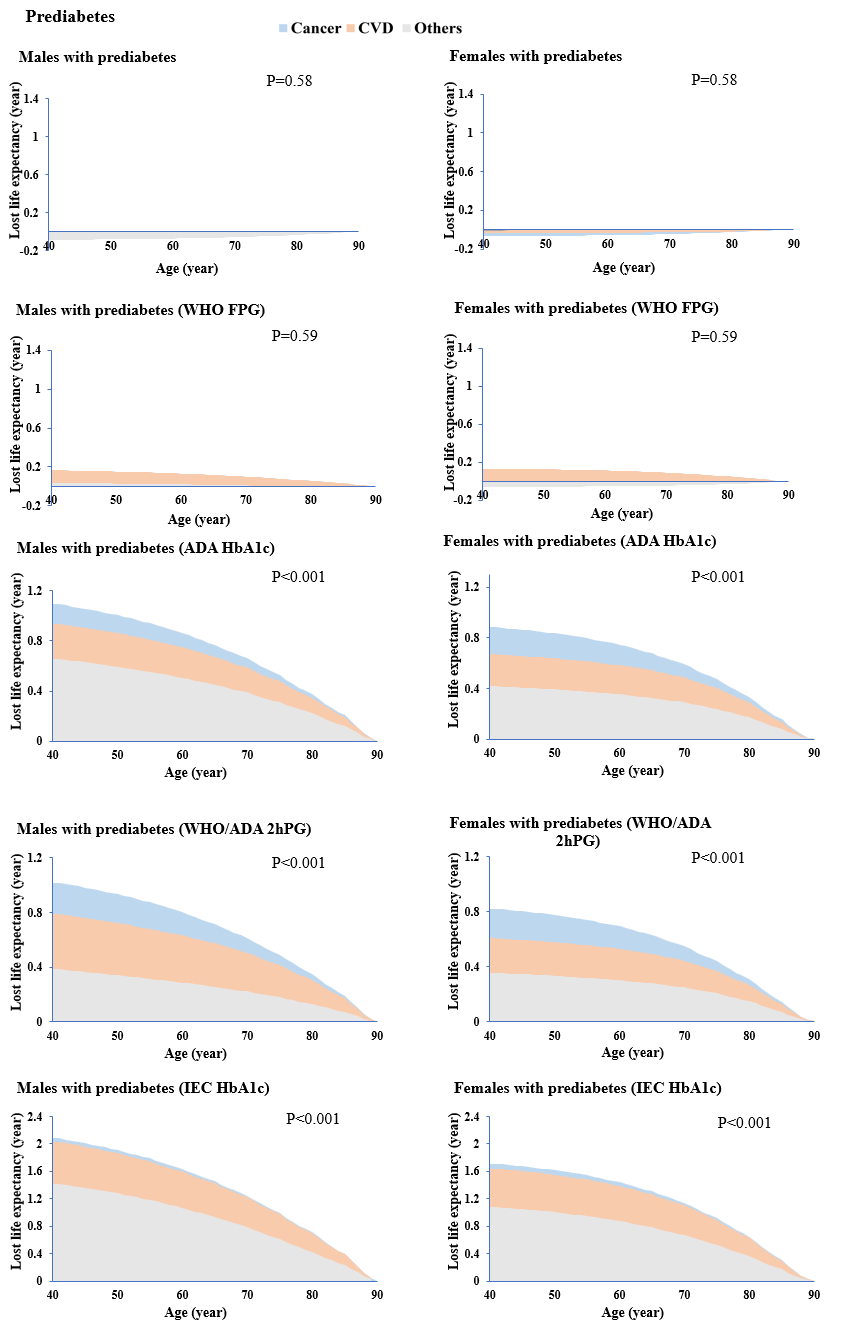


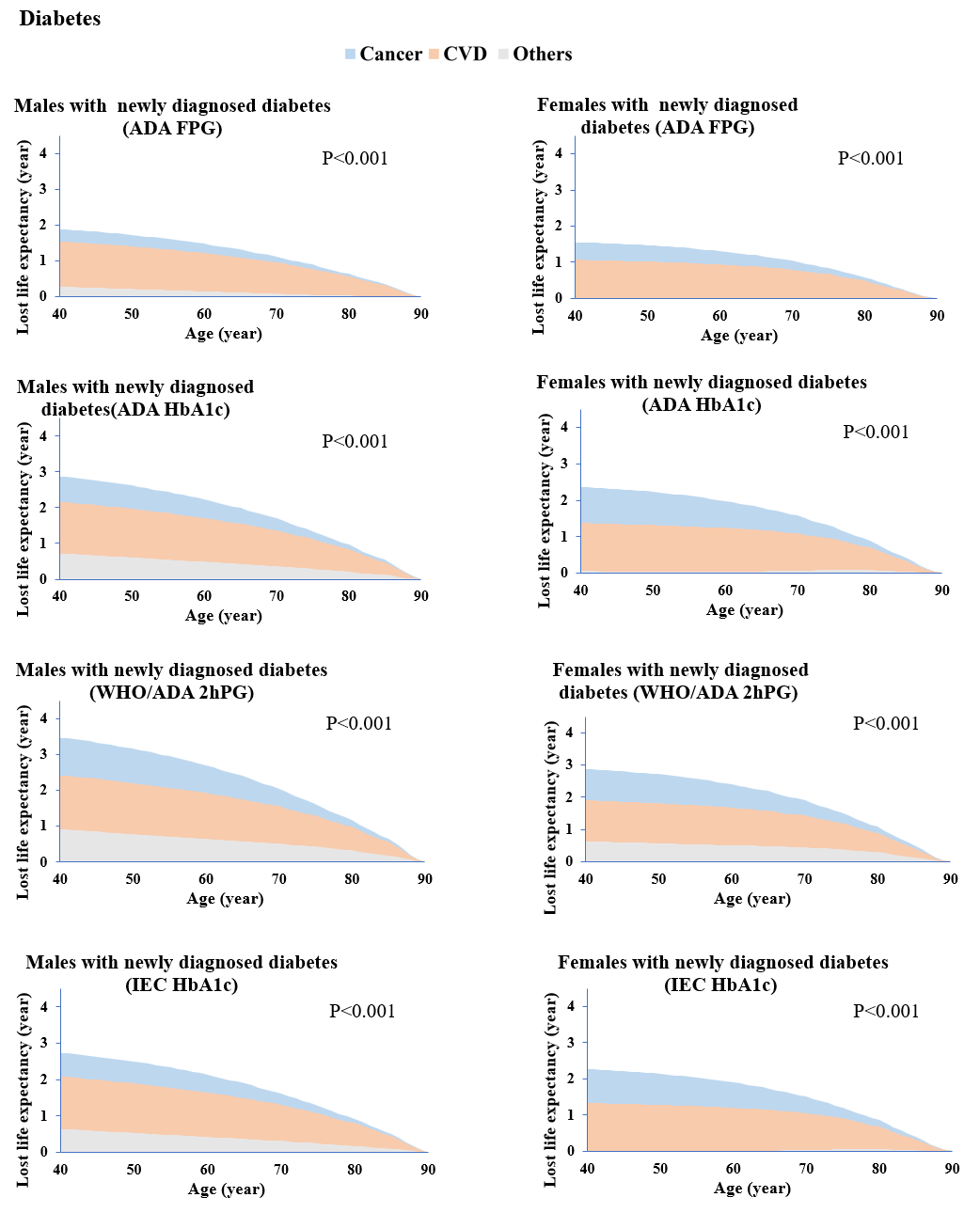


**Figure S5.** Sensitivity analysis of years of life lost due to cause-specific mortality using age-restricted life tables and competing-risk models.

The HRs applied to estimate life expectancy were adjusted for age (five-year group), sex (male, or female), residence (urban, or rural), education (junior high school and below, high school, college and above), household income (<¥20,000/year, ≥¥20,000/year, or not answer/don’t know), smoking (never smoked, past smoker, or active smoker), alcohol consumption (excessive, rare/nondrinker), physical activity (<150 min/week, or ≥150 min/week), red meat intakes (<100 g/day, or ≥100 g/day), vegetable and fruit intakes (<400 g/day, or ≥400 g/day), body mass index (<18.5, 18.5-23.9, 24-27.9, ≥28 kg/m^2^), hypertension (yes, or no), dyslipidemia (yes, or no) and self-reported CVD (yes, or no) and cancer (yes, or no). FPG, fasting plasma glucose; 2hPG, 2-hour postload glucose.
